# Macrophages orchestrate antiviral defense and epithelial repair in a human iPSC-derived alveolar air-liquid interface

**DOI:** 10.1172/jci.insight.203042

**Published:** 2026-03-17

**Authors:** Declan L. Turner, Hannah Baric, Katelyn Patatsos, Sahel Amoozadeh, Michael See, Kathleen A. Strumila, Jack T. Murphy, Jeremy J. Wiyana, Liam Gubbels, Elizabeth S. Ng, Andrew G. Elefanty, Melanie R. Neeland, Shivanthan Shanthikumar, Sarah L. Londrigan, Mirana Ramialison, Fernando J. Rossello, Ed G. Stanley, Rhiannon B. Werder

**Affiliations:** 1 Murdoch Children’s Research Institute, Melbourne, Victoria, Australia.; 2 Novo Nordisk Foundation Centre for Stem Cell Medicine, reNEW Melbourne, Melbourne, Australia.; 3 Department of Paediatrics, University of Melbourne, Melbourne, Australia.; 4 Respiratory and Sleep Medicine, Royal Children’s Hospital, Melbourne, Australia.; 5 Department of Microbiology and Immunology, The University of Melbourne at The Peter Doherty Institute for Infection and Immunity, Melbourne, Australia.; 6 Australian Regenerative Medicine Institute, Monash University, Clayton, Victoria, Australia.

**Keywords:** Cell biology, Infectious disease, Inflammation, Human stem cells, Macrophages, iPS cells

## Abstract

The lung alveoli are continually exposed to inhaled pathogens and environmental hazards and rely on coordinated communication between alveolar macrophages and type 2 alveolar epithelial cells (AT2s) to maintain homeostasis. Disruption of these interactions can impair immunity and repair, contributing to acute and chronic respiratory diseases. To better define these mechanisms and support therapeutic discovery, we established a human iPSC-derived air-liquid interface platform that captures key features of AT2-macrophage crosstalk. Using this system, we show that coculture enhances AT2-specific transcriptional programs including lipid synthesis, while macrophages actively phagocytose AT2-derived surfactant. iPSC-derived macrophages adopt an alveolar macrophage–like phenotype and respond to AT2-derived M-CSF. During respiratory infection, macrophages play a crucial role in modulating epithelial inflammatory responses, augmenting antiviral immunity, and limiting viral replication. We further identify a role for macrophages in epithelial repair, where VEGF-mediated signaling to macrophages increases epithelial permeability during viral infection. Together, these findings reveal dimensions of AT2-macrophage cooperation in homeostasis, infection, and repair, and demonstrate how this iPSC-derived platform can be used to dissect mechanisms that may initiate or drive the progression of respiratory diseases.

## Abstract 2

Human stem-cell-based lung model was developed to investigate how immune cells and alveolar cells work together to fight infection, and coordinate repair lung damage.

## Graphical abstract

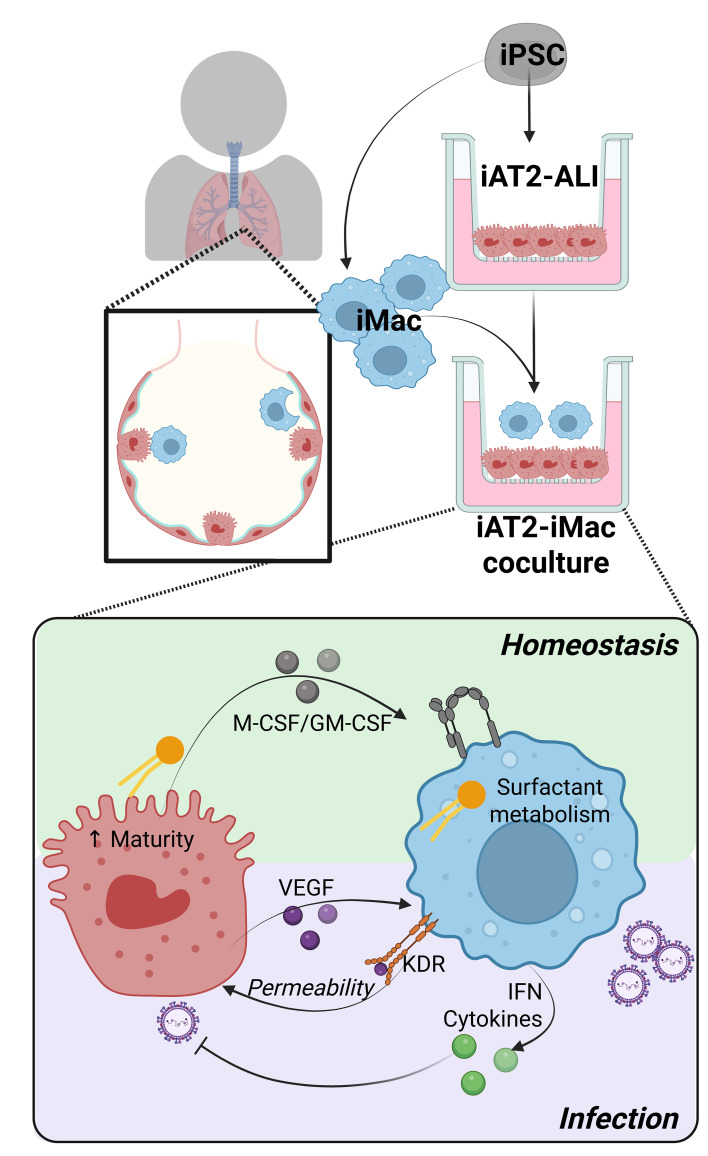

## Introduction

The lung alveoli are constantly exposed to inhaled pathogens and pollutants, requiring robust defense mechanisms. When these defenses are dysregulated, lung diseases can arise from persistent injury and abnormal repair. Alveolar macrophages act as frontline immune cells, clearing harmful particles and promoting tissue repair (1, 2); type 2 alveolar epithelial cells (AT2s) support immune regulation, surfactant production, and epithelial repair (3, 4).

Respiratory infections carry the highest disease burden, exceeding major public health threats like cancer and heart disease, as measured by disability-adjusted life years (5). Alveolar macrophages have a critical role in resolving respiratory viral infections in mice (6–9). AT2s are common targets for respiratory viruses and primary producers of infectious virus (10–12), and dysfunction and death of AT2s is associated with respiratory failure (13–15). Given the close proximity of alveolar macrophages and AT2s in the alveoli, studies have explored how immune responses to respiratory viral infections are influenced through cell crosstalk (16–18). For instance, AT2-derived surfactant protein A and surfactant protein D inhibit macrophage activation, in part through binding to SIRPα on alveolar macrophages (19–22). During influenza infection in mice, epithelial cells suppress alveolar macrophage activation through CD200 (23), which suggests that CD200R is critical for maintaining alveolar macrophage homeostasis. However, the expression of these ligand-receptor pairs discovered in mice differs in the human lung (LungMAP; refs. 24, 25). Furthermore, the inability to access alveolar tissue in vivo has restricted studies of human alveolar infection to late disease stages, typically after fatal cases (26). Thus, the study of respiratory viral infections and the discovery of new antivirals will necessitate the development of a human platform that incorporates the alveolar epithelium and macrophages.

The application of induced pluripotent stem cells (iPSCs) to create disease-relevant human cells is transforming the study of respiratory diseases. We and others have developed protocols using defined conditions to derive iPSC-derived AT2s (iAT2s) and macrophages (iMacs) (27–30). Crucially, these mimic in vivo functions, including surfactant biosynthesis (29) and immune function (30, 31), and therefore can be applied to successfully model genetic (29, 32, 33), acquired (34–36), and infectious (30, 31) lung diseases. Moreover, we have demonstrated that the reproducibility and scalability of these iPSC-derived platforms support drug screening (30, 37). To model AT2-macrophage interactions with iPSCs, previous work has introduced iMacs to alveolar organoids (38–40). However, because these organoids are polarized “apical-in,” they do not readily permit the study of respiratory infections and lack the native air-interface of the lungs, which we have previously shown to induce iAT2 maturation (34).

Here, we developed a human iPSC-derived platform incorporating AT2s and macrophages in an air-liquid interface (ALI) to study respiratory viral infections and repair. We demonstrated that coculture promoted maturation and function of both iAT2s and iMacs. After viral infections, iMacs heavily influenced proinflammatory signaling and antiviral immunity, which limited viral replication. Finally, we found that iMacs influenced epithelial repair through VEGFA/VEGFR2 signaling. This human model system has the potential to improve disease modeling and drug screening for various acute infections and chronic conditions affecting the lung alveoli.

## Results

### Establishment of iPSC-derived AT2s and macrophage coculture at ALI.

**Figure 1:**
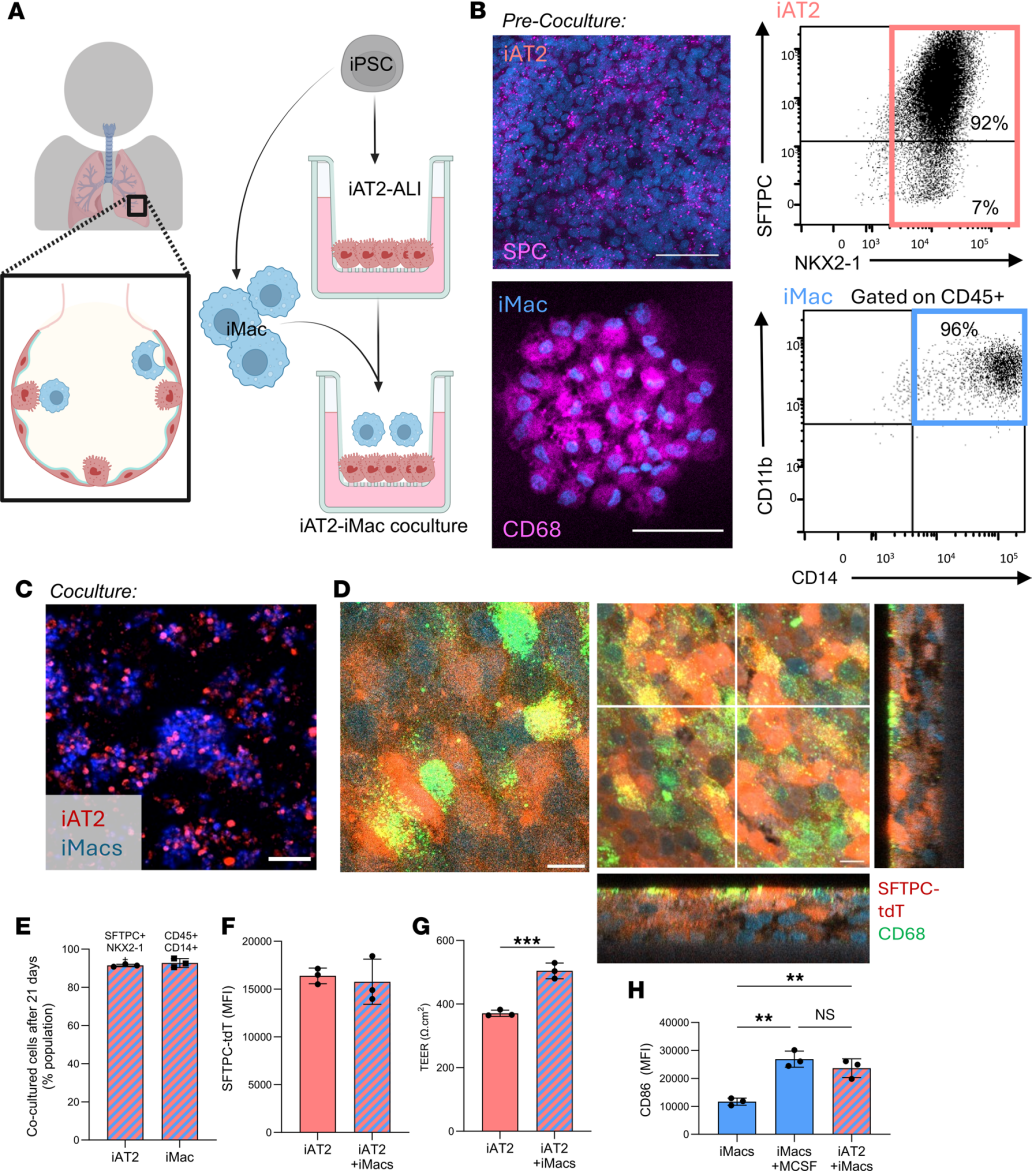
Establishment of iPSC-derived AT2 and macrophage air liquid interface cocultures. (**A**) Schematic representation of the coculture system. Separate iAT2 and iMac differentiations were performed. iAT2s were matured at air-liquid interface (ALI) for 3–7 days, then iMacs added to the apical compartment. (**B**) Prior to coculture, iAT2s expressed surfactant protein C, SFTPC-tdTomato, and NKX2-1*-*GFP. iMacs express CD68, CD14, and CD11b. Scale bar: 50 μm. (**C**) Live cell confocal imaging of iAT2 (red, marked by SFTPC-tdTomato) and iMacs (blue, stained with CellTrace violet) in coculture at ALI (48 hours after addition of iMacs). Scale bar: 100 μm. (**D**) Confocal imaging of iAT2s (red, SFTPC-tdTomato) and iMacs (green, CD68); scale bar: 10 μm, nuclei (blue) (13 days after addition of iMacs). (**E**) Percentage marker retention of iAT2s and iMacs after 21 days of coculture. iAT2s maintained expression of SFTPC-tdTomato and NKX2-1-GFP; iMacs expressed CD45 and CD14. (**F**) MFI of SFTPC-tdTomato in iAT2 alone or iAT2s after 7 days of iMac coculture. (**G**) Transepithelial electrical resistance (TEER) in iAT2 alone or iAT2s cocultured with iMacs for 7 days. (**H**) MFI of CD86 after 7 days cultured in CK-DCI, CK-DCI + M-CSF, or cocultured with iAT2s in CK-DCI. *n* = 3 experimental replicates of independent wells of a differentiation; data shown as mean ± SD. Statistical significance was determined by unpaired, 2-tailed Student’s *t* test (2 groups) or 1-way ANOVA (>2 groups); **P < 0.005, ***P < 0.001.

Human alveolar tissue is inaccessible in vivo. Given this, to develop an advanced model of the human alveolus, we constructed a coculture system that combined iAT2s and iMacs in the context of a physiologically relevant ALI culture system. Since each cell type arises from distinct germ layers and regions of the embryo, there are no established differentiation protocols to concurrently derive both cell types. Thus, to ensure our model contained pure, well-characterized cell populations, we separately differentiated iAT2s (27, 29) and iMacs (30), using previously published defined conditions (Figure 1A and Supplemental Figure 1A; supplemental material available online with this article; https://doi.org/10.1172/jci.insight.203042DS1). As expected, prior to coculture, iAT2s expressed NKX2-1 and surfactant protein C (28, 29), and iMacs expressed CD14, CD11b, and CD68 (30) (Figure 1B). To establish cocultures, iAT2s were matured in an ALI culture, as we have previously described (27, 31, 34), prior to the addition of macrophages in the apical compartment (Figure 1A). Cocultures were maintained solely in iAT2 media (termed CK-DCI). Flow cytometry analysis revealed the presence of both iAT2s and iMacs in cocultures (Supplemental Figure 1B). Live cell imaging of cocultured iAT2s (based on endogenous SFTPC-tdTomato ; ref. 29) and iMacs (labeled with CellTrace dye) demonstrated dynamic movement, viability, and longevity of iMacs in the cocultures (Figure 1C and Supplemental Video 1). iAT2-ALIs formed a multilayered epithelium, as has been shown with primary AT2s at ALI (41), with confocal imaging indicating a close association between iAT2 and iMacs, in which CD68^+^ macrophages were localized to the apical surface only (Figure 1D). We found that a similar approach could be employed to coculture iAT2s and primary alveolar macrophages from cryopreserved bronchoalveolar lavage fluid at ALI (Supplemental Figure 1C). To investigate whether an analogous epithelial-macrophage coculture could be established with AT1 cells, we created iAT1 cultures at ALI (42). Compared with iAT2-iMac cocultures, we observed very few adhered iMacs to the iAT1s (Supplemental Figure 1, D and E).

To assess the effect of coculture on the identity of either cell type, cocultures were dissociated 3 weeks after establishment and cells analyzed by flow cytometry for AT2 and macrophage markers. Both iAT2s (SFTPC^+^ NKX2-1^+^) and iMacs (CD45^+^ CD14^+^) retained their original identity (Figure 1E). iMacs comprised 25% of the culture at 1 week after addition, whereas after 3 weeks, iMacs comprised 1% of total cell numbers, compared with the initial seeding proportion of 10% (Supplemental Figure 1F). iAT2 expression of SFTPC-tdTomato was not significantly altered between cells in iAT2 monoculture versus cocultures (Figure 1F). Interestingly, barrier integrity, measured by transepithelial electrical resistance (TEER), was significantly enhanced by coculture with iMacs (Figure 1G and Supplemental Figure 1G). Levels of the costimulatory receptor CD86 were upregulated on cocultured iMacs (Figure 1H and Supplemental Figure 1, H and I), which was comparable to that observed with iMacs derived from cultures supplemented with M-CSF, an essential cytokine for iMac differentiation (30). Nearly all iMacs expressed the established alveolar macrophage markers, CD206 and CD169 (43), prior to coculture (Supplemental Figure 1J), and coculture therefore did not increase their expression (Supplemental Figure 1K). Collectively, these data demonstrate the successful establishment of iAT2-iMac cocultures at ALI, preserving cell identity while enhancing the functional properties of both cell types.

Although the alveolar macrophages at birth arise from primitive hematopoiesis, these are gradually replaced by monocyte-derived macrophages, which result from definitive hematopoiesis (44, 45). Numerous protocols describing the generation of iMacs have been established (46), with primitive- versus definitive-derived iMacs displaying some functional differences in vitro (47). Since the iMacs we had used thus far arose from a protocol that excludes retinoids and is therefore likely to generate macrophages mirroring those derived from extraembryonic (primitive) hematopoiesis (30), we also sought to examine the characteristics of macrophages generated with a protocol that supported the development of intra-embryonic (definitive) hematopoietic stem/progenitor cell intermediates (48, 49). iMacs derived through primitive and definitive routes both responded to respiratory syncytial virus (RSV) infection, upregulating IFN-stimulated genes (ISGs; *IFIT2* and *MX1*), although definitive iMacs displayed a higher magnitude of response late in infection (Supplemental Figure 2, A–C). We next created iAT2-iMac cocultures using iMacs derived from definitive hematopoiesis. As observed with our cocultures containing primitive iMacs, cocultures formed with definitive iMacs maintained their identity for 7 days (Supplemental Figure 2, D and E). Taken together, these findings suggest that the route of iMac differentiation does not substantially affect the establishment of cocultures, and subsequent experiments utilized primitive iMacs.

### iPSC-derived macrophages promote surfactant gene expression by iAT2s.

**Figure 2:**
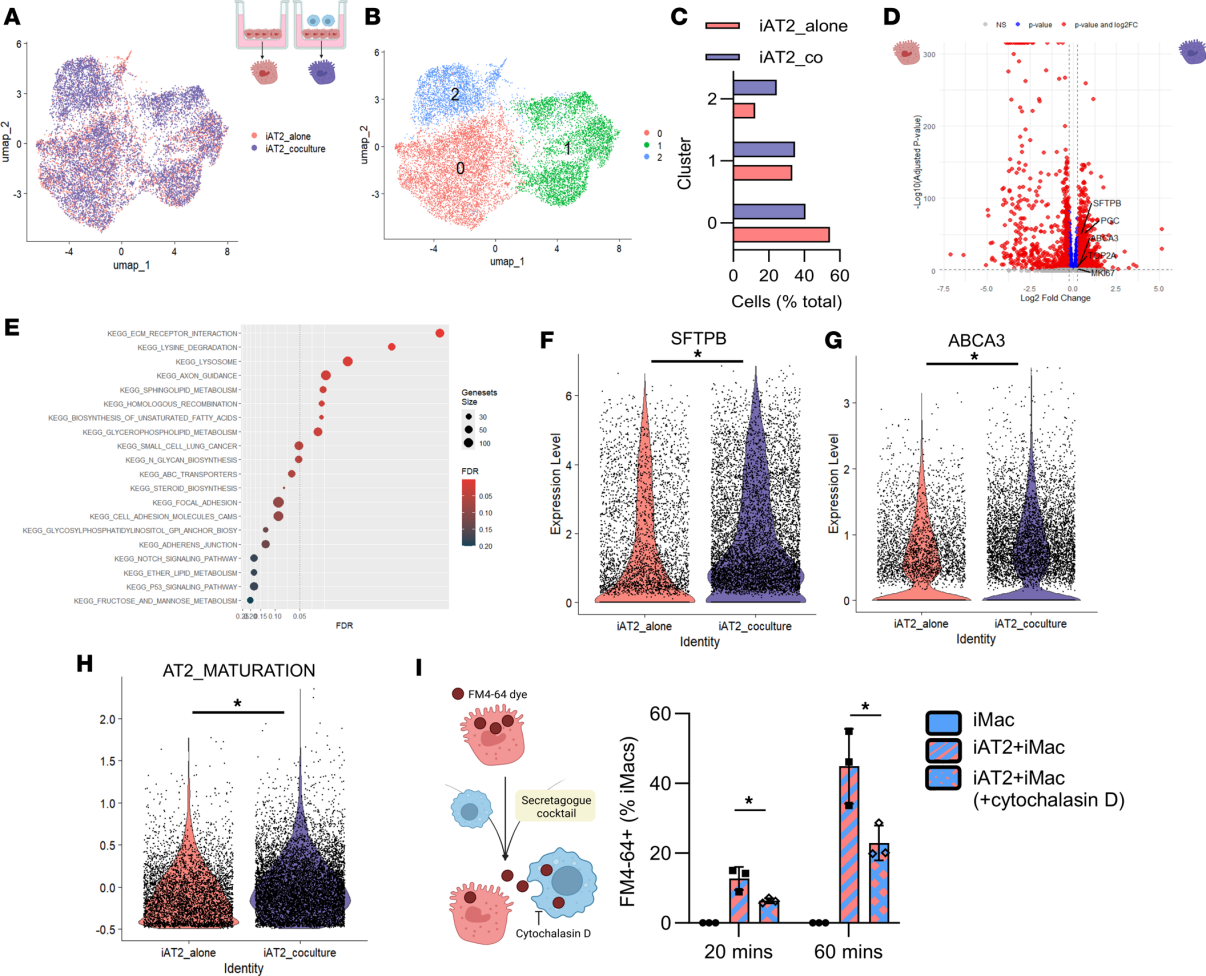
Coculture with iMacs promotes iAT2 transcriptional signature. (**A**) UMAP of iAT2 cells alone (pink) and iAT2 cells after coculture with iMacs (purple) for 5 days. (**B**) Louvain clustering at a resolution of 0.1. (**C**) Proportion of iAT2 alone (pink) or iAT2 after coculture (purple) in each cluster at Louvain resolution 0.1. (**D**) Volcano plot of differentially expressed genes upregulated in iAT2 alone (pink, left) or iAT2 after coculture (purple, right). (**E**) Gene set enrichment analysis depicting upregulated pathways in iAT2s after coculture compared with iAT2s alone. (**F**) Violin plots of differentially expressed genes including glycosylated surfactants *SFTPB (*log_2_FC = 0. 41, adjusted *P* = 1.65 × 10^–14^) and (**G**) surfactant transporter *ABCA3* (log_2_FC = 0.37, adjusted *P* = 9.35 × 10^–5^). Statistical significance for **F** and **G** determined by Wilcoxon rank-sum test. (**H**) Module score of AT2 maturation gene set (45) indicating that coculture enhances AT2 maturation. Statistical significance was determined by Welch’s 2-sample *t* test. (**I**) iAT2s were treated with the lipophilic dye FM4-64, washed, and then treated with a secretagogue cocktail (ATP and PMA). iMacs were immediately added and incubated for 20 or 90 minutes prior to collection and flow cytometry to measure internalized FM4-64 in iMacs. iMacs were treated with cytochalasin D to inhibit phagocytosis. Statistical significance was determined by 2-way ANOVA. *n* = 3 experimental replicates of independent wells of a differentiation; data shown as mean ± SD. Statistical significance tests indicated for each panel; *P < 0.05.

Surfactant homeostasis in the lung is maintained through synthesis and recycling by AT2s and degradation by alveolar macrophages (50). Surfactant production is the hallmark function of AT2s in vivo, and iAT2s robustly recapitulate this process, producing and secreting surfactant proteins and lipids packaged within lamellar bodies (Supplemental Figure 3A) and enabling extensive studies of intrinsic surfactant dysfunction (29, 32, 33). However, no existing model has yet incorporated the contribution of macrophages to maintaining surfactant homeostasis, to our knowledge. In initial experiments, expression of *SFTPA1*, a marker of iAT2 maturation (51), was unchanged 3 days after coculture but was significantly increased 5 days after the addition of iMacs (Supplemental Figure 3B). To explore how coculture with iMacs would influence the transcriptome of iAT2s, we performed single-cell RNA-Seq (scRNA-Seq) of monocultures and cocultures (Figure 2A and Supplemental Figure 3, C–F). iAT2s were established in air for 2 days prior to the addition of iMacs for 5 days. Uniform manifold approximation and projection (UMAP) visualization suggested large overlap of transcriptomes of iAT2s alone and cocultured iAT2s (Figure 2A), although unsupervised cell clustering (Louvain) revealed a cluster was predominated by cocultured iAT2s (cluster 2) (Figure 2, B and C). As we had observed in preliminary studies, coculturing iAT2s did not adversely alter expression of key AT2 markers (e.g., *SFTPA1, SFTPC*), nor promote the emergence of non–lung endoderm, off-target lineages (Supplemental Figure 3D). Indeed, differential gene expression analysis revealed an upregulation in AT2 genes (*PGC, SFTPB, ABCA3*) and proliferation genes (*MIKI67, TOP2A*) in cocultured iAT2s compared with iAT2s alone (Figure 2D). Gene set enrichment analysis revealed that pathways involved in lipid synthesis and metabolism, glycosylation, and ATP-binding cassette transporters were upregulated in iAT2s after coculture with iMacs (Figure 2E and Supplemental Figure 3, G–L), suggestive of changes in surfactant synthesis and processing. N-linked glycosylation is a key posttranslational modification, essential for the secretion and function of surfactant proteins (52, 53). Intriguingly, expression of the surfactant proteins that undergo glycosylation, (e.g., *SFTPB*) was upregulated in a subset of cocultured iAT2s (Figure 2F). Furthermore, *ABCA3*, the essential surfactant lipid transporter on lamellar bodies, was differentially upregulated in iAT2s after iMac coculture (Figure 2G). Quantification of AT2 differentiation and maturation gene modules generated from primary AT2s (51) demonstrated that maturation was significantly increased in iAT2s in cocultures compared with iAT2s alone (Figure 2H and Supplemental Figure 3M). Collectively, our single-cell transcriptomic profiling suggested that a proportion of iAT2s increase surfactant production and packaging in the presence of iMacs, potentially as a consequence of surfactant metabolism by the macrophages. To confirm that iMacs were taking up surfactant in our model, we labeled lipids with a lipophilic dye and then stimulated iAT2s to secrete surfactant, as previously described (33). Flow cytometry revealed that iMacs rapidly and efficiently phagocytosed extracellular lipids secreted by iAT2s (Figure 2I and Supplemental Figure 3, N and O). Importantly, this was not due to cell death of iAT2s (Supplemental Figure 3P). Furthermore, inhibition of iMac phagocytosis significantly reduced uptake of lipophilic dye (Figure 2I), suggesting that iMacs were metabolizing surfactant in our iAT2-iMac coculture system, mirroring the activity of their in vivo counterparts.

### iPSC-derived macrophages are supported through iAT2-derived M-CSF.

**Figure 3:**
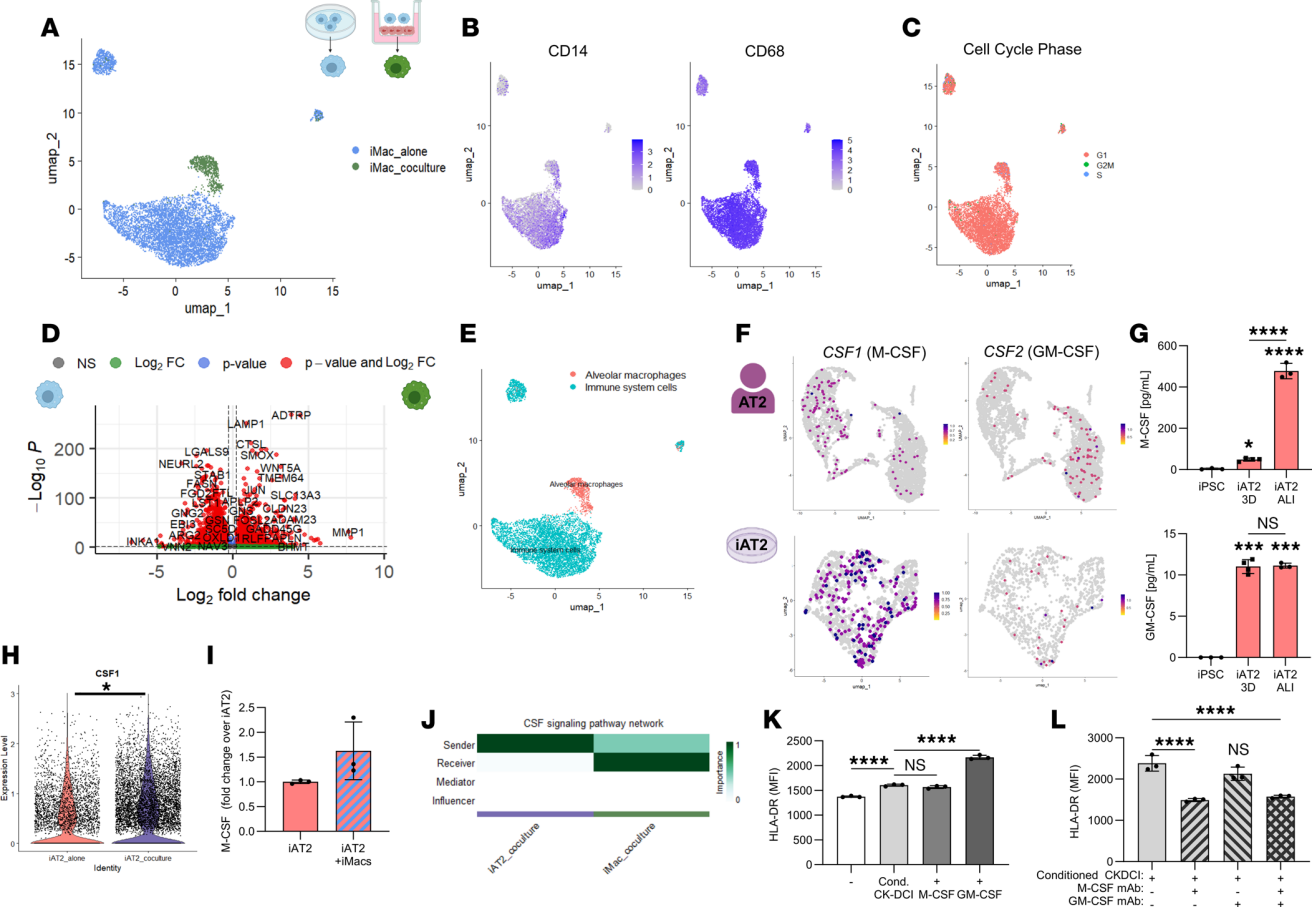
iMacs are sustained in coculture by iAT2-derived M-CSF. (**A**) UMAP of iMacs alone (blue) or iMacs after coculture with iAT2s (green) for 5 days. Populations clustered distinctly based on original identity. (**B**) UMAPs showing expression of *CD14* and *CD68*. (**C**) UMAP of cell cycle phase. (**D**) Volcano plot of differentially expressed genes upregulated in iMac alone (left) or iMac after coculture (right). (**E**) scType analysis of iMacs alone or iMacs after coculture with iAT2s. (**F**) *CSF1* and *CSF2* expression in adult human AT2s (97) and iAT2s (32, 33). (**G**) M-CSF and GM-CSF secretion was measured by ELISA from undifferentiated iPSCs, iAT2s in 3D conditions, and iAT2s at ALI. Statistical significance was determined by 1-way ANOVA; compared with iPSCs or as indicated. (**H**) *CSF1* expression in iAT2 alone (pink) or iAT2 after coculture with iMacs (purple). *CSF1* expression was increased in cocultured iAT2s. Statistical significance was determined by Wilcoxon rank-sum test (log_2_FC = 0.37, adjusted *P* = 1.37 × 10^–6^). (**I**) M-CSF secretion was measured in the basolateral compartment of iAT2 or iAT2-iMac cocultures at ALI. Levels normalized to iAT2 alone. (**J**) The CSF signaling pathway network identified by CellChat analysis of iAT2s in coculture (purple) or iMacs in coculture (green). (**K**) Conditioned CK-DCI from iAT2s was added to iMacs alone. Alternatively, iMacs were cultured in CK-DCI supplemented with M-CSF or GM-CSF. MFI of HLA-DR was measured by flow cytometry after 72 hours. Statistical significance was determined by ANOVA with Tukey’s post hoc test. (**L**) iMacs treated with conditioned CK-DCI from iAT2s were incubated with neutralizing antibodies against M-CSF, GM-CSF, or both. HLA-DR MFI was measured by flow cytometry after 72 hours. Statistical significance was determined by 1-way ANOVA with Tukey’s post hoc test; *P < 0.05, ***P < 0.001, ****P < 0.0001.

We next sought to examine the consequences of coculture on iMacs. scRNA-Seq revealed that cocultured iMacs cluster discretely from monocultured iMacs (Figure 3, A and B). Coculture did not cause iMacs to enter cell cycle (Figure 3C), yet coculture of iMacs did significantly alter their transcriptome compared with iMacs alone (Figure 3D). To compare cocultured iMacs to in vivo cell types, we used scType for cell-type identification (54). Cocultured iMacs were most similar to primary human alveolar macrophages compared with iMacs cultured alone, which were classified broadly as immune system cells in the lung (Figure 3E). In light of the tissue resident–like identity that cocultured iMacs adopted, we next sought to understand the influence of iAT2s in this process. In the adult human lung, AT2s produce M-CSF and GM-CSF (55) (Supplemental Figure 4A), and we observed that iAT2s expressed similar transcript levels of both cytokines (Figure 3F). Moreover, we demonstrated that homeostatic iAT2 secreted M-CSF and GM-CSF protein (Figure 3G). Strikingly, iAT2s cultured at ALI secreted significantly more M-CSF than when maintained in their standard 3D format (Figure 3G), highlighting the greater maturity and physiological relevance of the ALI system (34). In the presence of iMacs, iAT2s further upregulated transcript expression and protein levels of M-CSF (encoded by *CSF1*) and to a lesser extent GM-CSF (encoded by *CSF2*) (Figure 3, H and I, and Supplemental Figure 4, B and C). We next assessed cell-cell interactions using CellChat (56) between cocultured iAT2s and iMacs. Among the significantly enriched signals from iAT2s to iMacs was *CSF1-CSF1R* (Figure 3J and Supplemental Figure 4D), suggesting that iMacs may be sustained in coculture by iAT2-derived M-CSF. Given that M-CSF is soluble, we reasoned that iMac maintenance would be independent of contact with iAT2s. To test this, we collected conditioned media from iAT2 cultures and used this to supplement isolated iMac cultures. Conditioned media did not alter iMac proliferation but significantly upregulated HLA-DR expression, to a similar extent as iMacs maintained in M-CSF–containing media (Figure 3K and Supplemental Figure 4E). Of note, GM-CSF–supplemented iMacs expressed significantly higher levels of HLA-DR, consistent with enhanced activation mediated by GM-CSF in previous reports (57, 58). To understand whether iAT2-derived M-CSF was directly responsible for iMac activation, we used neutralizing antibodies to block M-CSF, GM-CSF, or both in iAT2-conditioned media. Blockade of M-CSF or both M-CSF/GM-CSF, but not GM-CSF alone, inhibited the upregulation in HLA-DR expression in iMacs (Figure 3L), mirroring the effect elicited by simply removing exogenous M-CSF from iMacs (Supplemental Figure 4F). Collectively, our data suggest that iMacs are maintained in cocultures by iAT2-derived M-CSF.

### iPSC-derived macrophages promote inflammation and innate antiviral immunity after respiratory viral infection of iPSC-derived type 2 alveolar epithelium.

**Figure 4:**
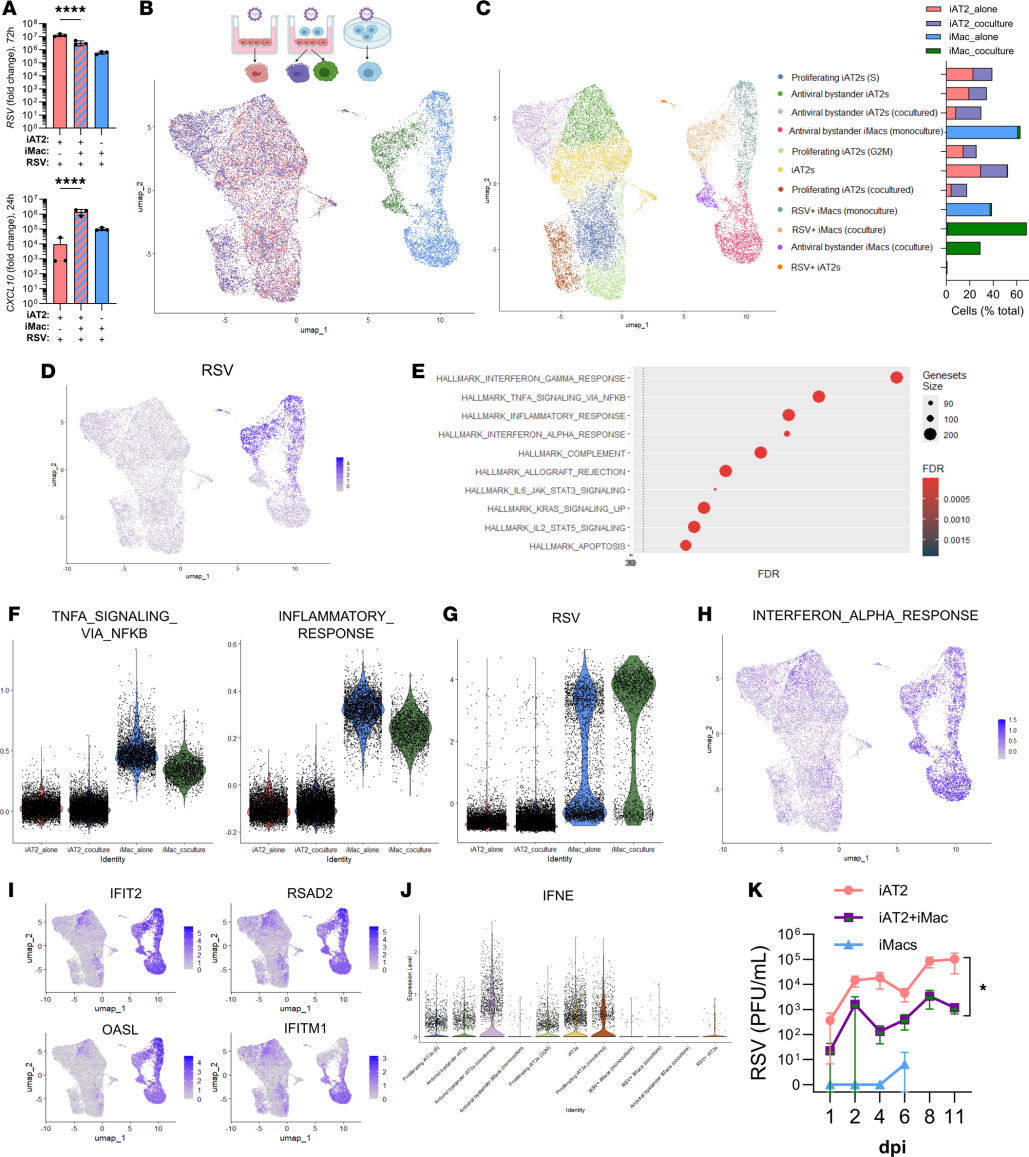
iMacs augment inflammation and antiviral immunity during viral infections of cocultures. (**A**) qRT-PCR for *RSV N* and *CXCL10* expression after RSV (MOI 1) infection of iAT2 alone, iAT2-iMac cocultures, or iMac alone. (**B**) scRNA-Seq analysis of iAT2 alone, iMac alone, and cocultures 48 hours after RSV (MOI 10) infection. UMAP of cells showing original identity. (**C**) Louvain clustering and proportion of each population to each cluster. (**D**) UMAP with module score (whole genome) of RSV transcript expression. (**E** and **F**) Gene sets enriched in iMacs after coculture from the Hallmark database. (**G**) Module score (whole genome) of RSV transcript expression in each population. (**H**) UMAP with module score of “Interferon Alpha Response” from the Hallmark database highlighted. (**I**) Feature plots of select IFN-stimulated genes highlighting distinct responses between RSV-infected populations. (**J**) *IFNE* expression in Louvain clusters (resolution 0.4, **C**). (**K**) Shed RSV collected in apical washes from iAT2 alone or iAT2-iMac cocultured ALIs over 11 days. Infectious RSV was titered by plaque assay, *n* = 3, data shown as mean ± SD, statistical significance was determined by 2-way ANOVA with Tukey’s post hoc test; *P<0.05, ****P < 0.0001.

Although most respiratory viruses preferentially replicate in the upper respiratory tract, these viruses can spread to the lower respiratory tract and alveolus, causing severe infections like pneumonia. In the alveolus, human-tropic respiratory viruses like RSV and influenza A (IAV) commonly target AT2s (10, 11). Because human alveolar tissue cannot be readily accessed in vivo, the early stages of infection remain largely uncharacterized, with available data derived from late or fatal disease. To test our coculture platform in the context of infection, we treated cultures with the viral mimetic poly(I:C) or infected the cultures with IAV or RSV. After exposure, iAT2-iMac cocultures expressed significantly higher antiviral IFN-λ and antiviral ISGs (*CXCL10* and *MX1*), compared with iAT2s alone or iMacs alone (Figure 4A and Supplemental Figure 5, A–D). To explore this further, we performed scRNA-Seq 48 hours after RSV infection of iAT2s or iMacs alone, or iAT2-iMac cocultures (Figure 4B and Supplemental Figure 5, E–H). UMAP visualization and cell clustering revealed that iMacs segregated from iAT2s after infection. Moreover, cocultured iMacs clustered distinctly from iMacs alone (Figure 4, B and C). Although this division was less defined in the iAT2s, we did observe certain clusters predominantly contained iAT2s from cocultures (Figure 4C and Supplemental Table 6). Three clusters contained RSV transcripts, representing actively infected iMacs alone, infected iMacs from cocultures, and infected iAT2s (Figure 4, C and D). Previous studies in mice have shown alveolar macrophages are key producers of type I IFNs (59), the proinflammatory cytokines TNF, IL-1α, IL-1β, IL-6, and chemokines (e.g., CCL2–4) (60, 61) during RSV infections. Supporting the fidelity of our coculture system, iMacs were the primary producer of these antiviral cytokines, proinflammatory cytokines, and chemokines (Supplemental Figure 5, I and J). Furthermore, unbiased gene set enrichment analysis revealed that inflammatory pathways were enriched in iMacs compared with iAT2s (Figure 4, E and F). Overall, while these pathways were upregulated in both iMacs alone and in coculture, cocultured iMacs appeared less inflammatory yet enriched for more viral transcripts (Figure 4, F and G), suggesting the coculture environment shapes iMac immune responses.

Innate antiviral immunity is a vital defense against respiratory viruses through the production of type I and III IFNs, which induce ISGs that directly inhibit viral replication or recruit immune cells. We found that iAT2s infected with RSV or IAV did not produce type I IFN (IFN-β), and only IAV but not RSV elicited type III IFN (IFN-λ2). By contrast, iMacs mounted a robust IFN-β response (Supplemental Figure 5, K and L). This was supported in our scRNA-Seq data where innate IFN responses, including ISGs, were more enriched in iMacs compared with iAT2s after infection (Figure 4, H and I). Interestingly, expression of certain ISGs (e.g., *IFITM1*) were largely restricted to bystander iMacs and iAT2s (i.e., cells that did not express RSV transcripts in virus-infected cultures). Although actively infected iAT2s were rare, we sought to understand whether coculture with a professional immune cell would reshape antiviral immunity in iAT2s. Notably, certain clusters of bystander iAT2s, but not the actively infected iAT2s, were enriched for IFN signaling (Figure 4, H and I). Significant enrichment for type I IFN (*IFNE*) as well as IFN receptors (*IFNLR1*) and downstream signaling molecules (*TYK2*) was observed in the bystander cluster predominantly containing cocultured iAT2s (Figure 4, C and J, and Supplemental Figure 5M). Notably, we observed similar rates of initial iAT2 infection between mono and cocultures (Supplemental Figure 5N). This indicates that in cocultures, iMacs augment antiviral immunity, suggesting that viral replication and shedding may be partially constrained in this platform. To investigate this, we infected iAT2 or iAT2-iMac ALIs with RSV and measured shed infectious virus over 11 days. RSV release was almost entirely absent in the control infected iMacs alone, which was unsurprising given the epithelium is the major target, and consistent with previous reports showing abortive infection of RSV in alveolar macrophages (62), although these cells died after 6 days (Figure 4K and Supplemental Figure 5O). Virus continued to shed in iAT2 cultures over 11 days, and this was significantly reduced in cocultures with iMacs (Figure 4K), consistent with enhanced antiviral immunity restraining RSV replication.

### iPSC-derived AT2 repair and permeability is influenced by macrophages.

**Figure 5:**
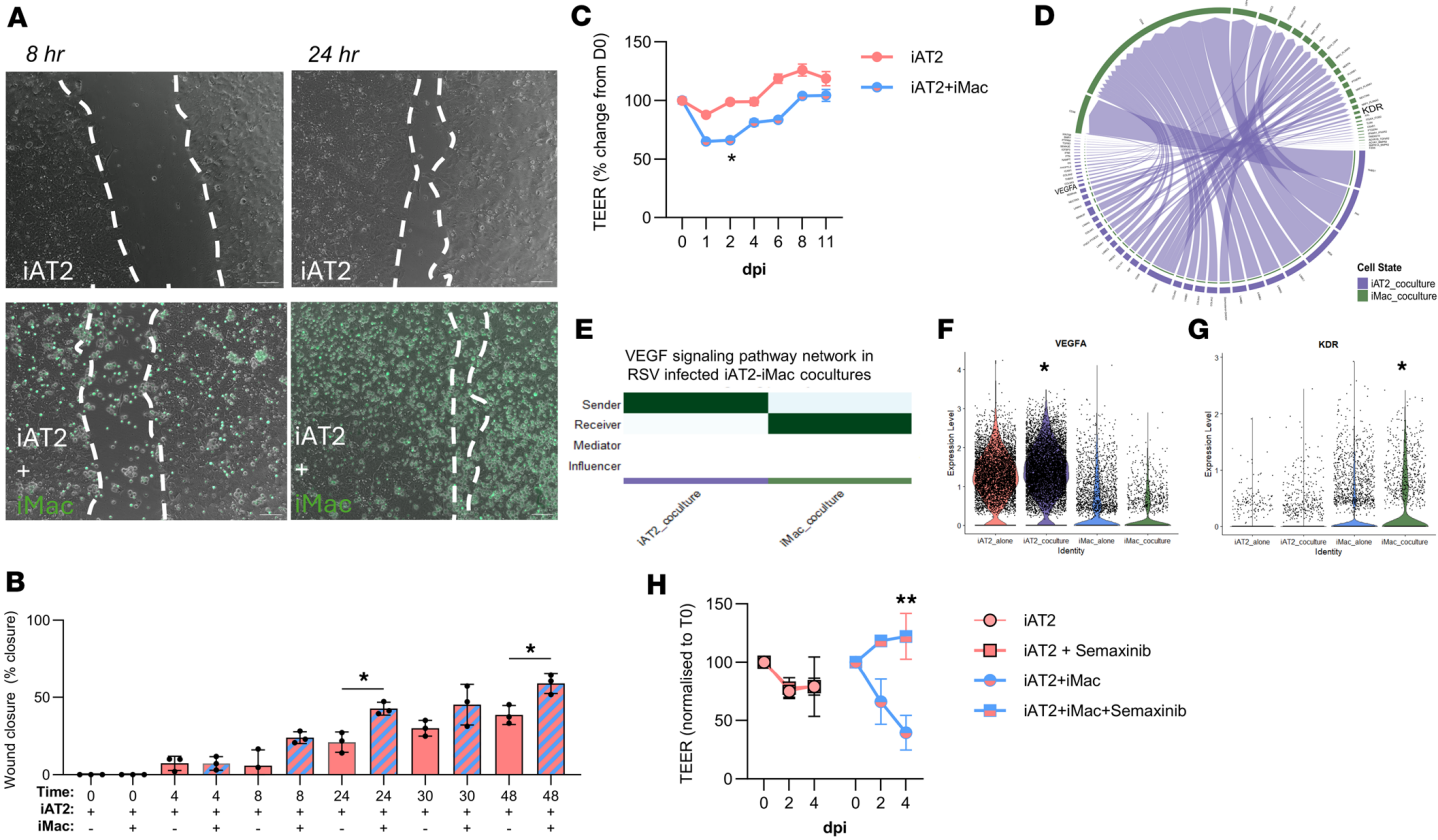
iMacs influence iAT2 repair through VEGF signaling. (**A** and **B**) iAT2s were plated in 2D and allowed to reach confluence before a scratch wound was made. iMacs were labeled with CFSE (green) and added immediately after the scratch wound. Wound closure was calculated as a percentage of the initial wound over a 48-hour period. Scale bar: 100 μm. (**C**) Transepithelial electrical resistance (TEER) normalized to preinfection of ALIs containing iAT2s alone or iAT2-iMac cocultures infected with RSV (MOI 10) over 11 days. (**D**) Circos plot showing CellChat analysis of iAT2 signals (purple) to iMacs (green) in RSV-infected cocultures. (**E**) VEGF signaling pathway identified in CellChat analysis of iAT2s in coculture (purple) or iMacs in coculture (green). (**F**) VEGFA and (**G**) KDR (VEGFR2) expression in RSV-infected iAT2s alone, iAT2s from coculture, iMacs alone, or iMacs from coculture, showing iAT2s are the predominant source of VEGFA during RSV infection, and cocultured iMacs upregulate KDR. Statistical significance determined by Wilcoxon rank-sum test. (**H**) TEER normalized to preinfection of ALIs containing iAT2s alone or iAT2-iMac cocultures infected with RSV (MOI 10), then treated with semaxanib (KDR inhibitor) in the basolateral compartment. *n* = 3 experimental replicates of independent wells of a differentiation; data shown as mean ± SD. Statistical significance was determined by 1-way (**B**) or 2-way ANOVA (**C** and **H**); *P < 0.05, **P < 0.005.

Alveolar macrophages play critical roles in wound healing after lung infections by stimulating proliferation of structural cells (epithelium and fibroblasts), promoting angiogenesis, and dampening inflammation (63). To explore whether iMacs would influence wound healing, we performed a scratch assay through a monolayer of iAT2s (36) with or without iMacs, and then monitored wound closure. iMacs significantly accelerated iAT2 wound closure over a 48-hour period (Figure 5, A and B, and Supplemental Figure 6A). Since respiratory viruses can injure the epithelium through disrupted tight junctions and cytotoxicity (64, 65), we also assessed whether iMacs would change TEER during RSV infection of iAT2s. Interestingly, early in infection (2 days after infection), the presence of iMacs impaired TEER (Figure 5C and Supplemental Figure 6B). To investigate pro- or anti-repair mechanisms engaged by epithelial-macrophage crosstalk, we assessed cell-cell communication at 2 days after RSV infection in our scRNA-Seq dataset. Infection prompted 6 new ligand-receptor interactions, which were not present between iAT2s and iMacs in uninfected conditions (Supplemental Figure 6, C–E). Among these infection-only pathways, the VEGFA/VEGFR2 axis was the only one in which the damaged iAT2s acted as senders, expressing *VEGFA*, while iMacs served as direct receivers via VEGFR2 (also known as *KDR*), forming a linear ligand-receptor interaction (Figure 5, D–G). In the homeostatic human lung in vivo*,* type 1 alveolar epithelial cells (iAT1s) are the predominant source of VEGFA, although low expression is evident both in primary AT2s and iAT2s (Supplemental Figure 6, F and G). Strikingly, iMacs further upregulated *KDR* expression during coculture compared with iMacs alone (Figure 5G). VEGF signaling plays varied roles during infection and in recovery from lung injury, acting as a chemotactic agent, mitogen, and angiogenic factor (66–70). Whether VEGF has predominantly beneficial or detrimental effects, particularly in the context of human AT2 cells and macrophages during respiratory viral infections, is unknown. To investigate this, we inhibited VEGFR2/KDR signaling using semaxanib (SU5416) (71). RSV infection impaired TEER in iAT2s alone, and this was unaltered by semaxanib treatment (Figure 5H and Supplemental Figure 6G). As we had observed previously (Figure 5C), TEER significantly declined in iAT2 cultures containing iMacs. However, by 4 days after infection, increased barrier permeability was entirely blocked during RSV infection by inhibiting VEGFR2/KDR signaling in cocultures (Figure 5H and Supplemental Figure 6H). Given that semaxanib does not target viral replication, it was unsurprising that viral shedding was unaffected by treatment (Supplemental Figure 6I). Together, this suggests that early during infection, VEGFA/VEGFR2 signaling between iAT2s and iMacs alters epithelial permeability.

## Discussion

Many acute and chronic respiratory diseases directly affect the alveoli. The development of human model systems that recapitulate interactions between key cell types will be critical for the discovery of new therapeutics. In this study, we establish an iPSC-derived platform that incorporates AT2 cells and macrophages in a physiologically relevant ALI culture system, which is easily amenable to infection studies. Coculture upregulated AT2-specific genes and lipid synthesis in iAT2s and iMacs phagocytosed surfactant. iAT2s supported iMacs in coculture through the production of M-CSF, and iMacs adopted an alveolar macrophage–like phenotype. Importantly, iMacs promoted proinflammatory signaling and antiviral immunity, and they limited viral replication during respiratory viral infections. Additionally, we found that iMacs influenced epithelial barrier repair and integrity, in part through VEGFA/VEGFR2 signaling.

Recent studies have described the incorporation or codevelopment of macrophages in iPSC-derived organotypic models, including the gut and brain (72, 73). In these models, iMacs acquire transcriptional signatures resembling tissue-resident macrophages, regulate immune signaling, and promote organoid maturation (40, 72, 73). Mirroring these observations, iMacs in our model adopted an alveolar macrophage transcriptional signature, enhanced proinflammatory signaling and antiviral immunity during viral infections, and supported iAT2 maturation.

When establishing our model, we found that exogenous macrophage-supportive factors were unnecessary since iAT2s alone could sustain iMacs. During lung development, the alveolar epithelium arises concurrently with alveolar macrophage differentiation (45, 74), and alveolar epithelial cells remain a major source of GM-CSF and M-CSF into adulthood (55). iAT2s express both *CSF1* and *CSF2* at levels comparable to in vivo human AT2s and secrete both cytokines at baseline, with M-CSF levels further upregulated at ALI. Of note, other macrophage-supportive cytokines like IL-3 and IL-34 were not expressed by iAT2s. iAT2-derived M-CSF appeared crucial to the maintenance of iMacs. We previously demonstrated that M-CSF alone is sufficient to induce and sustain functional iMacs (30), consistent with the ability of iAT2-derived M-CSF to maintain a stable population of iMacs within cocultures. It is important to note that GM-CSF is indispensable for the development and survival of alveolar macrophages in mice (45, 55, 75), whereas M-CSF–deficient mice exhibit reduced alveolar macrophage numbers, which can be compensated by other cytokines (76). Thus, although M-CSF signaling appeared critical for the iAT2-iMac cocultures, we cannot entirely exclude a role for iAT2-derived GM-CSF.

Respiratory viruses commonly cause pneumonia in infants, and RSV is a leading cause of pneumonia cases, hospitalizations, and mortality in this age group (77). RSV is a human-restricted pathogen, with minimal replication evident in small animal models (78). Furthermore, since respiratory viruses enter through the apical side of the epithelium, ALI models (but not organoids) support robust infection (79). To our knowledge, this is the first study to describe RSV infection in an ALI model that recapitulates the biology of primary AT2s, using both iAT2s alone and iAT2s with iMacs. Previous AT2 studies have primarily focused on other human-specific viruses such as coronaviruses (31, 80). In the absence of in vivo datasets, our model provides a valuable platform to investigate early infection responses in the alveolus that cannot otherwise be studied in humans. Surprisingly, despite productive and sustained infection, our scRNA-Seq data revealed very few iAT2s were actively infected at 48 hours, although the epithelium is the target of RSV. Furthermore, iAT2s produced little IFN and only subsets of iAT2 bystanders activated antiviral defenses, such as IFN-ε and ISGs, in contrast to RSV-infected airway epithelial cells, where antiviral responses appear more uniform (81). It would be interesting in future studies to assess whether a gradient of iAT2 bystander antiviral responses to RSV is determined by proximity to an infected cell (82).

During viral infections in mice, alveolar macrophage depletion can either lead to respiratory failure (6) or improve survival (7, 8), suggesting potentially virus-specific effects and underscoring the delicate balance between inflammation, antiviral immunity, cell death, and repair. In our cocultures, iMacs were the primary infected cell type, aligning with findings from bronchoalveolar lavage studies of RSV-infected infants (83). Moreover, the presence of iMacs in cocultures significantly reduced viral replication. iMacs likely impaired viral burden through both abortive infection (62) and through prompting augmented antiviral immunity in iAT2s. This reiterates the importance of incorporating innate immune cells into respiratory epithelial models to faithfully recapitulate the sequelae of infection.

Alveolar epithelial damage during viral infections plays a key role in pneumonia pathology and can lead to severe complications such as acute respiratory distress syndrome (13, 14). We demonstrated that iMacs promote iAT2 repair in both a scratch assay and during RSV infection. In mice, several macrophage-derived ligands (e.g., Wnt and IL-1β) have been implicated in AT2 proliferation, differentiation, and recovery from injury (84, 85). Our scRNA-Seq analysis identified WNT signaling between iMacs and iAT2s at baseline and during infection; however, we were unable to determine any potential role that this axis may play in repair due to the presence of a GSK3β inhibitor in the media, which is necessary for iAT2 maintenance (29). Interestingly, VEGF-A/VEGFR2 signaling emerged between iAT2s and iMacs only after viral infection, increasing barrier permeability early in infection. Although macrophages are typically a source of VEGF in other organs, AT1s and to a lesser extent AT2s are the main producers in the lungs (24, 25), explaining *VEGFA* expression patterns in our cocultures. VEGF-C/VEGFR3 signaling is known to regulate macrophage functions such as efferocytosis during acute lung injury (86); however, this appears to be the first report of VEGF-A from human AT2s signaling through VEGFR2 on macrophages during a respiratory viral infection. Future studies should explore whether clinically used VEGF-targeting therapies, such as small molecules and monoclonal antibodies employed in cancer treatment, influence respiratory viral infection outcomes.

The development of iAT2-iMac ALI cocultures is an important first step toward more closely aligning the complexity of in vitro alveoli models with their in vivo counterparts. Ideally, these models will extend to incorporating other cell types, including AT1 cells. However, despite recent progress in generating pure iAT1 cells (42), current methods are unable to generate populations of iAT2s and iAT1s at frequencies that yield the proportion of each cell type present within the native alveolus. Similarly, our model could be further elaborated to include other lung-resident immune cells, such as DCs, innate lymphoid cells, and tissue-resident lymphocytes. However, as a starting point, our iPSC-derived platform serves as a human in vitro model for studying AT2-macrophage interactions in homeostasis, infection with human-tropic respiratory viruses, and repair. Access to this model should facilitate disease modeling by providing insights into the relative contributions of AT2s and alveolar macrophages to respiratory disease initiation and progression.

## Methods

### Sex as a biological variable.

Both male and female iPSC lines were utilized, and similar findings are reported for both sexes.

### Human iPSC maintenance.

Human iPSCs were cultured in StemFlex (Thermo Fisher Scientific) or mTeSR1 or TeSR-E8 (Stemcell Technologies) on plates coated with Matrigel (Corning, 354277). Experiments were conducted in multiple iPSC lines, which have been previously described: BU3 NGST CRISPRi (36), SCT3010 (MCRIi032-A, RRID: CVCL_D0I2; Stemcell Technologies/MCRI), PB001 (87), and PiMM1 and PiMM6 (88, 89).

### Directed differentiation of iAT2s.

Directed differentiation of iAT2s was performed as we have previously described (27, 29). In brief, iPSCs were differentiated to CXCR4^+^ cKit^+^ definitive endoderm using the STEMdiff Definitive Endoderm kit (Stemcell Technologies). Cells were then dissociated with Gentle Cell dissociation reagent (Stemcell Technologies), replated on growth factor–reduced Matrigel-coated plates (Corning, 354277), and cultured in anteriorization media for 3 days (complete serum-free differentiation medium [cSFDM] as base media, supplemented with 2 µM dorsomorphin [Tocris, 3093/10] and 10 µM SB431542 [Tocris, 1614]). To induce NKX2-1^+^ lung progenitors, cells were moved to cSFDM supplemented with 3 µM CHIR99021 (R&D Systems, RDS442310), 10 ng/mL recombinant human BMP4 (R&D Systems, 314-BP), and 100 nM retinoic acid (Sigma-Aldrich, R2625). On day 14–15, cells were dissociated with 0.05% trypsin (Thermo Fisher Scientific), and NKX2-1^+^ lung progenitors were purified (based on NKX2-1-GFP or CD47^hi^CD26^lo^) by FACS using a FACSAria Fusion (BD Biosciences). Sorted NKX2-1^+^ lung progenitors were embedded in growth factor–reduced Matrigel (Corning, 356230) droplets and supplemented with 3 μM CHIR99021, 10 ng/mL rhKGF (R&D Systems, RDS251KG050), 50 nM dexamethasone (Sigma-Aldrich, D4902), 0.1 mM 8-bromoadenosine 30,50 cyclic monophosphate sodium salt (Sigma-Aldrich, B7880), and 0.1 mM 3-isobutyl-1methylxanthine (IBMX; Sigma-Aldrich, I5879) in cSFDM (CK-DCI media). CK-DCI media was replaced every 2–3 days. iAT2s were serially passaged every 2 weeks and resorted when needed based on NKX2-1-GFP^+^ SFTPC-tdTomato^+^ expression or carboxypeptidase M (CPM) positivity, as described (27, 36). After each passage, cultures initiated with single cells were supplemented with 10 µM Y-27632 (‘‘Y’’; Tocris, RDS125410) in CK-DCI media. After 2–3 days in Y-27632, medium was replaced with CK-DCI alone.

To create ALI cultures, iAT2s were dissociated using 0.05% trypsin to generate a single-cell suspension. Next, 200,000 cells were plated onto growth factor–reduced Matrigel-coated 6.5 mm Transwells (Costar) in CK-DCI media, as described (27, 34). The apical media was removed to create an ALI 2–3 days later. Basolateral media was changed every 2–3 days. To create iPSC-derived iAT1s, 200,000 iAT2s were plated on 6.5 mm Transwells in cSFDM supplemented with 10 µM LATS-IN-1 (MedChemExpress, HY-138489), 50 nM dexamethasone, 0.1 mM 8-bromoadenosine 30,50 cyclic monophosphate sodium salt, and 0.1 mM IBMX (L-DCI media), as described (42). TEER measurements were taken with a Millicell ERS-2 Voltohmmeter (MilliporeSigma, MERS00002). The electrodes were submerged in 100 μL PBS in the apical compartment and 500 μL CK-DCI in the basolateral chamber. Average values were calculated from 3 recordings taken at different locations within the Transwell.

### Directed differentiation of iPSC-derived macrophages.

Directed differentiation of iMacs was performed as we have previously described (30). In summary, on day 0 iPSCs were dissociated and resuspended in Magec media (30) supplemented with 20 ng/mL VEGF (R&D Systems, 293-VE), 20 ng/mL SCF (synthesized by CSIRO, Australia), 5 ng/mL FGF2 (R&D Systems, RDS233FB500), 10 ng/mL BMP4 (R&D systems, 314-BP), 10 μM Y-27632, 0.5 μM CHIR99021, and 10 ng/mL activin A (R&D Systems, 338-AC). To form embryoid bodies (EBs), cells were cultured in non-tissue culture–treated dishes (Greiner Bio-One, 628161) on a Ratech rotating platform at 60 rpm in a 37°C incubator, as described (90). On days 1 and 3, the media was changed by allowing EBs to settle without centrifugation, then aspirating media and replacing with Magec media supplemented with 20 ng/mL VEGF, 20 ng/mL SCF, 10 ng/mL FGF2, and 10 ng/mL BMP4. From day 6 to 13, media was supplemented with 20 ng/mL VEGF, 20 ng/mL SCF, 10 ng/mL FGF2, and 25 ng/mL M-CSF (PeproTech, 300-25), and from day 13 onward comprised 50 ng/mL M-CSF. After day 9 or 10, media changes were performed by centrifuging at 300*g* for 5 minutes to pellet EBs. Cells were filtered through a Falcon 40 μm cell strainer (Corning, 35240) at day 15–16 to remove EBs; then, iMacs were analyzed by flow cytometry and used for experiments over the next 2–3 weeks. Media was replaced twice weekly by centrifuging at 300*g* for 5 minutes to pellet cells, and then media containing 50 ng/mL M-CSF was replenished.

To create a source of “definitive” iMacs, we first created iPSC-derived hematopoietic stem cells (iHSCs), as previously described (48). At day 14 of this protocol, iHSCs were plated in 50 ng/mL M-CSF and 5 ng/mL SCF in SPELS media (48) in non-tissue culture–treated dishes on a Ratech rotating platform at 60 rpm in a 37°C incubator. Media was replaced every 2–3 days. After 1 week, media was changed to 50 ng/mL M-CSF in SPELS and cultured for another 3 weeks. Media was replaced twice weekly by centrifuging at 300*g* for 5 minutes to pellet cells. The cell pellet was then resuspended in fresh media and returned to the incubator.

In some experiments, iMacs were cultured in low-attachment 96-well plates in CK-DCI media, supplemented with 50 ng/mL M-CSF or 50 ng/mL GM-CSF (PeproTech, 300-03). Alternatively, CK-DCI media conditioned by 2–3 days dwelling in 3D iAT2 cultures was added to iMacs. To block M-CSF or GM-CSF signaling, conditioned media cultures were treated with 4 μg/mL anti-M-CSF and/or anti-GM-CSF (R&D Systems, RDSMAB216SP and RDSMAB615SP).

To visualize live macrophages in cultures, iMacs were labeled with CellTrace CFSE or violet at room temperature for 20 minutes, per the manufacturer’s instructions (Thermo Fisher Scientific, C34554 and C34557).

### Coculture of iAT2 and iMacs.

To establish iAT2-iMac cocultures at ALI, iAT2s were seeded in Transwells, as described above. Next, 4–7 days after initiation of ALI, 20,000 iMacs were added to the apical surface of each Transwell resuspended in 5–10 μL CK-DCI (this volume was reabsorbed or evaporated in 2–3 days). Experiments were conducted 4–21 days after the addition of iMacs, as indicated. A 10:1 ratio of iAT2/iMacs was selected based on the estimated frequency of iAT2s and alveolar macrophages in the human lung (91, 92).

### Flow cytometry and cell sorting.

Endoderm cells were stained for CXCR4 (BD Biosciences, 555974) and c-Kit (BioLegend, 313206) and analyzed using a Fortessa flow cytometer (BD Biosciences). NKX2-1^+^ cells were isolated on the basis of NKX2-1-GFP expression or expression of CD47 and CD26. For the latter, cells were stained with antibodies (CD47-PerCPCy5.5, 323110 and CD26-PE 323110, BioLegend) for 30 minutes on ice, and then CD47^hi^/CD26^lo^ cells isolated by FACS (28). Where indicated, SFTPC^+^ iAT2s were purified by FACS on the basis of a SFTPC-tdTomato reporter gene (29) or on the basis of CPM expression (Novachem, 014-27501) (34). Cells were resuspended in sort buffer (HBSS, Thermo Fisher Scientific, 2% FBS, 10 mm Y-27632). Live cells were sorted using 10 mM Calcein blue AM (Life Technologies) and Zombie NIR or Zombie R718 (BioLegend, 423106 and 423115). Cells were isolated using a FACS Aria (BD Biosciences) at the Murdoch Children’s Research Institute (MCRI) Flow Cytometry Core Facility.

To assess iMac differentiation or activation, cells were stained with CD45-BV421 (BioLegend, 304032), CD14-PECy7 (BioLegend, 301814), HLA-DR-FITC (BD Biosciences, 347363), CD86-APC (BioLegend, 305412), CD206-APC-Cy7 (BioLegend, 321119), CD169-BV421 (BioLegend, 346017), and CD11b-APC (BD Biosciences, 550019) antibodies. iMac proliferation was measured by CFSE dilution using flow cytometry per the manufacturer’s instructions (Thermo Fisher Scientific, C34554). Live cells (assessed by Calcein blue AM or Zombie) were analyzed on a Fortessa (BD Biosciences).

To assess intracellular RSV infection, cells were stained with Zombie dye, then fixed and permeabilized (Foxp3 Fixation/Permeabilization solution, eBioscience 00-5523-00) per the manufacturer’s instructions. Cells were incubated with primary antibody (RSV F, Abcam, ab94968), then secondary antibody (anti-mouse APC, BD Biosciences 550826) diluted in 1× permeabilization buffer.

### Immunostaining, confocal imaging, and transmission electron microscopy.

Samples were fixed with 4% PFA (Santa Cruz Biotechnology, sc281692) for 20 minutes at room temperature and stored at 4°C prior to staining. ALI membranes were excised with a scalpel blade prior to immunostaining. Samples were permeabilized with 0.3% Triton X-100 (Sigma-Aldrich, T8787) and blocked with 4% normal donkey serum or normal goat serum (Sigma-Aldrich). Blocking solution was used to dilute primary antibodies, which were incubated at 4°C overnight. Samples were washed prior to incubation with fluorescently conjugated antibodies and counterstained with DAPI (Sigma-Aldrich, D9542) for 1 hour at room temperature. Live cell imaging or immunostaining were imaged with a LSM 900 confocal microscope (Zeiss) and images were processed using ImageJ (NIH). Antibodies used in this study were surfactant protein C (Santa Cruz, sc518029), CD68 (Abcam, ab213363), HT1-56 (Terrace Biotech, TB-29AHT1-56), ZO-1 (Invitrogen, 61-7300), EpCAM (Abcam, ab7504), and RSV (Abcam, ab43812 and Merck, AB1128). Fluorescently conjugated secondary antibodies were purchased from Invitrogen.

For transmission electron microscopy, iAT2s on Transwells were fixed in 2.5% glutaraldehyde followed by 2% osmium tetroxide, embedded in resin, and then sectioned (50–90 nm). Images were acquired on a JEM-1400 TEM (JEOL Ltd.) operating at 80 kV, taken using a 14mp NanoSprint AMT camera and the native AMT software.

### Viral isolation and infection.

RSV strain A2 (ATCC VR-1540) was propagated in Hep-2 cells as previously described (93). Briefly, Hep-2 cells were infected at low MOI and incubated at 37°C for 5 days in DMEM (Thermo Fisher Scientific, 21969035) supplemented with 10% FBS (Thermo Fisher Scientific, A5670701), 10 U/mL penicillin, and 10 U/mL streptomycin (Thermo Fisher Scientific, 15070063) (DMEM complete). Infected cells were scraped into the supernatant, then centrifuged at 1,500*g* for 10 minutes. Clarified supernatant was underlaid with 5 mL sucrose cushions (30% m/v sucrose, 1× PBS, pH 7.4) in SW28 ultracentrifuge tubes and the virus pelleted at 20,000*g* for 90 minutes at 4°C. Pellets were resuspended in DMEM without supplementation and aliquots snap-frozen on dry ice, and then stored at –80°C. Titers of RSV stocks or experimental samples were determined using an immuno-plaque assay. In brief, Hep-2 cells were seeded in 96-well culture plates and inoculated with serial 1:5 dilutions of stock/sample for 2 hours with occasional agitation, inoculum was removed, and then incubated for 3 days with a 1% methyl cellulose (Sigma-Aldrich, M7027) overlay in DMEM complete. Cells were fixed with 4% PFA for 15 minutes, permeabilized with 0.3% Triton X-100 for 15 minutes, and washed 3 times in PBS-T (0.1% Tween 20). Fixed plates were blocked with 4% BSA for 1 hour, incubated with anti-RSV antibody (1:500) (Merck, AB1128) for 90 minutes, washed as before, and then incubated with Alexa Fluor 488 conjugated secondary antibody (Thermo Fisher Scientific, A32790) for 1 hour. All steps were performed at room temperature. Plaques were manually counted on a fluorescent microscope (Zeiss Observer.Z1) at 10× original magnification, and titers calculated as PFUs per milliliter.

Influenza A Virus (A/PR8/34; H1N1) stocks were propagated in the allantoic cavity of 10-day embryonated chicken eggs through collaboration with the WHO Collaborating Centre for Reference and Research on Influenza (WHO CCRRI) at the Doherty Institute, Melbourne, and titered using MDCK cells by standard plaque assay, as previously described (94).

ALIs were infected apically with RSV-A2 (MOI 10 or 1) or IAV H1N1 (MOI 2) diluted in 50 μL DMEM. iMacs alone were cultured in tissue culture–treated 96-well plates for infection studies. Inoculum was removed after 2 hours, cells washed, then returned to air (ALIs) or media replenished (iMacs alone). ALI apical washes with PBS were taken every 2–3 days to harvest shed virus. TEER measurements were taken simultaneously. To inhibit VEGFR2/KDR, 10 μM semaxanib (SU5416) (MedChemExpress, HY-10374) was added to the basolateral compartment after the initial viral inoculum was removed and media replenished after 48 hours. In some experiments, instead of live viral infections, the apical compartment of ALIs were treated with 10 μg/mL poly(I:C) (InvivoGen, INV-tlrl-pic) diluted in OptiMem with Oligofectamine (Thermo Fisher Scientific, 12252011) for 24 hours.

### Quantitative real-time PCR.

RNA was extracted using the ISOLATE II RNA Mini kit (Bioline, BIO-52073) per the manufacturer’s protocol. cDNA was reverse transcribed using the Tetro cDNA Synthesis kit (Bioline, BIO-65043). Quantitative real-time PCR (qRT-PCR) was run for 45 cycles using PowerTrack SYBR Green Master Mix (Thermo Fisher Scientific, A46111) and custom primers (Table 1). For each biological replicate, the average Ct value for technical triplicates was calculated and normalized to the housekeeping gene (*ACTB*). Fold-change was determined using 2^ΔΔCt.

### ELISA.

Media was collected from the basolateral compartment of ALIs or from iPSCs, iAT2s in 3D Matrigel droplets (cultured as above), or iMacs cultured in tissue culture–treated 96-well plates. Media was immediately snap-frozen and stored at –80°C. Protein secretion was measured per the manufacturer’s instructions, using the following commercial ELISA kits: M-CSF (Invitrogen, EHCSF1), GM-CSF (Invitrogen, KHC2011), IFN-β (Invitrogen, 414101), and IFN-λ2/IL28A (R&D Systems, DY1587).

### scRNA-Seq.

scRNA-Seq was performed using the Flex Gene Expression assay (10x Genomics, 1000475). iAT2 ALIs were established at air, and in some Transwells iMacs were added to the apical surface, as described above. Three days later, half the Transwells were infected with RSV (MOI 10), as described above. iMacs alone were plated on 6-well tissue culture–treated plates in CK-DCI and infected as above. Uninfected or infected samples were dissociated 48 hours later using Accutase (Stemcell Technologies, 7922). To achieve sufficient cell numbers, Transwells from the same condition were pooled as appropriate. Cells were stained with Zombie R718 for 15 minutes at room temperature, and then incubated with anti-CD45 antibody. Cells were fixed with fixation buffer (10x Genomics, 1000475) for 21 hours. After quenching fixation as per the manufacturer’s instructions, Zombie-negative cells were sorted. In iAT2-iMac cocultures, CD45^–^ iAT2s and CD45^+^ iMacs were sorted separately, then pooled after sorting 10:1. Cells were collected from the sort in Lo-bind Eppendorf tubes supplemented with RNase inhibitor (Promega, M6101). Cells were counted, and then stored at –80°C, per the manufacturer’s recommendations.

After storage, monocultured iAT2s and iMacs, as well as infected iAT2s and iMacs, were pooled 1:1; then, all samples were hybridized with human WTA probes BC001-BC004 and 26 custom spike-in probe pairs against RSV A2 genes at 40 nM per probe for all samples (Supplemental Table 1). Custom probes were designed according to 10x Genomics tech note CG000621_RevC and purchased as standard desalted oPools from IDT and resuspended in low EDTA TE buffer (Thermo Fisher Scientific, 12090015). Samples were multiplexed prior to GEM generation using a Chromium iX (10x Genomics). GEMs were recovered and gene expression libraries constructed by following the manufacturer’s protocols. Libraries were sequenced on an Illumina NovaSeq X plus (AGRF).

More than 94% of sequencing generated reads had a quality score of 30 (Q30). scRNA-Seq bioinformatics analysis performed using the Cell Ranger pipeline (version 8.0.0) was used to create fastq files and count matrices. Seurat (v5) was used for further analysis and data visualization. Doublets and cells with more than 5% of reads mapping to mitochondrial genes were filtered out and data were normalized using SCTransform. UMAP and principal components analysis were used for dimensionality reductions and clusters determined by the Louvain algorithm. Cell-cycle stage was calculated as described (95). Monocultured iAT2s and iMacs as well as infected monocultured iAT2s and iMacs combined prior to barcoding were assigned unique identities based on Louvain clustering and expression of NKX2.1 greater than 1 and CD68 greater than 1, respectively, and analyzed as unique biological samples thereafter. Differentially expressed genes were determined using the FindAllMarkers function implemented in Seurat, with a Wilcoxon rank-sum test and a default log fold-change of 0.1. Gene set enrichment analysis was performed with hypeR using ranked differentially expressed gene lists (96). Cell-type identification was performed using scType (54). Cell-cell communication was inferred using CellChat (56). Data are deposited in NCBI’s Gene Expression Omnibus (GEO) under accession GSE294640.

Analysis of previously published datasets was performed to analyze expression of *VEGFA, CSF1,* and *CSF2* in primary AT2s and iAT2s. Violin plots of expression from lung epithelial cells used data generated by the LungMAP Consortium (24, 25), which was downloaded (www.lungmap.net) on November 5, 2025, and viewed using the ShinyCell application. The LungMAP consortium, the Human Tissue Core (U01-HL144861), and the LungMAP Data Coordinating Center (U24-HL148865) are funded by the National Heart, Lung, and Blood Institute (NHLBI). To generate UMAPs, healthy AT2s were subsetted based on expression of *SFTPC* (>4) from a previously published study of the human lung (97). Control iAT2s from previous studies (35, 36) were subsetted for further analysis here.

### Lipid uptake assay.

To label intracellular lipids, iAT2s were plated on Matrigel-coated plates (Corning, 354277), and then incubated with 5 μg/mL FM 4-64 dye (N-(3-triethylammoniumpropyl)-4-(6-(4-(diethylamino) phenyl) hexatriene) pyridinium dibromide) (Thermo Fisher Scientific, T13320) for 20 minutes. iAT2s were washed prior to addition of a “secretagogue cocktail” consisting of 100 nM ATP (Thermo Fisher Scientific, R0441) and 300 nM PMA (Abcam, AB147465). iMacs were immediately added. To inhibit phagocytosis, iMacs were pretreated with cytochalasin D (20 μM, Sigma-Aldrich, C2618) at 37°C for 30 minutes before addition to iAT2s. At 20 or 90 minutes later, cultures were dissociated with Accutase, stained for cell surface markers on ice, and analyzed on a Fortessa flow cytometer (as above).

### Wound healing assay.

First, 300,000 iAT2s were plated on Matrigel-coated 48-well plates (Corning, 354277) for 24 hours, prior to the addition of iMacs (10:1 ratio). A linear scratch through the iAT2 monolayer was introduced using a sterile P10 pipette tip (time 0) and imaged over 48 hours, as we have previously described (36). Wound area was quantified using ImageJ (NIH) software. The initial wound area at 0 hours was manually delineated and used as a fixed reference for subsequent time points. Wound closure was calculated as the percentage reduction in wound area relative to baseline. In coculture conditions, iMacs frequently occupied the wound space, which reduced visual contrast of the wound edge; however, epithelial wound boundaries remained identifiable based on continuous iAT2 monolayer morphology. Representative images are shown in Supplemental Figure 6A.

### Statistics.

Statistical analyses were performed using unpaired 2-tailed Student’s *t* tests for comparisons between 2 groups and 1-way ANOVA with a Tukey multiple-comparison test for comparisons among 3 or more groups. Details for each analysis are provided in the figure legends. Data are shown as mean ± SD. A *P* value of less than 0.05 was considered statistically significant.

### Study approval.

Ethical approval for the generation and/or use of human iPSCs was obtained from MCRI and Boston University, and experiments were carried out in accordance with the National Health and Medical Research Council of Australia (NHMRC) regulations.

### Data availability.

All data values are reported in the Supporting Data Values file. The scRNA-Seq data that support the findings of this study have been deposited in the NCBI’s GEO under accession GSE294640.

## Author contributions

RBW conceptualized the project; DLT, HB, and RBW designed experiments; DLT, HB, KP, SA, KAS, JTM, JW, and RBW performed experiments; DLT, LG, MN, SS, and RBW performed scRNA-Seq experiments; DLT, MS, FJR, and RBW performed bioinformatics analyses; SLL provided influenza virus; EN, AE, MR, FJR, and ES provided expert input on experimental design and data interpretation; RBW and DLT wrote the first draft of the manuscript. All authors critically reviewed and approved the final version of the manuscript.

## Conflict of interest

FJR receives institutional and salary support as a coinvestigator and subcontractor with the Peter MacCallum Cancer Centre for an investigator-initiated trial, which receives funding support from Regeneron Pharmaceuticals; and as a coinvestigator on a translational research project funded by a Regeneron Pharmaceuticals grant.

## Funding support

This work was supported by the following sources:

- Stafford Fox Medical Research Foundation.
- L.E.W. Carty Trust.
- Novo Nordisk Foundation Center for Stem Cell Medicine (grant NNF21CC0073729).

## Supplementary Material

Supplemental data

Supplemental table 1

Supplemental video 1

Supporting data values

## Figures and Tables

**Table 1:**
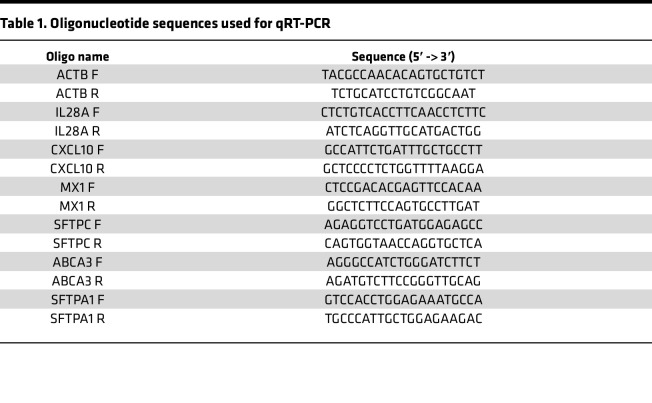
Oligonucleotide sequences used for qRT-PCR

## Version 1 03/17/2026

In-Press Preview

## Version 2 05/08/2026

Electronic publication
