# Supplementary material for: Macrophages orchestrate antiviral defense and epithelial repair in a human iPSC-derived alveolar air-liquid interface
Source: JCI Insight. 2026 Mar 17;11(9):e203042. doi: 10.1172/jci.insight.203042 (PMC13232486; doi:10.1172/jci.insight.203042)
Supplement: Supplemental data [file jciinsight-11-203042-s269.pdf]

SUPPLEMENTAL DATA

Position-5-Driven Reorientation of an Immunodominant HLA-A*24:02 SARS-CoV-2 Epitope Drives Universal T-cell Escape

Takeshi Nakama[1†], Aaron Wall[2†], Garry Dolton[2†], Li-Rong Tan[2], Hannah Thomas[2], Hiroshi Hamana[3], Yoshiki Aritsu[1], Toong Seng Tan[1], Mako Toyoda[1], Yoshihiko Goto[1], Huanyu Li[1], Mizuki Kitamatsu[4], Keiko Udaka[5], Yusuke Miyashita[6, 7], Hiroyuki Oshiumi[6], Kimitoshi Nakamura[7], Yoji Nagasaki[8], Rumi Minami[9], Hirotomo Nakata[10], Pierre J Rizkallah[2], Hiroyuki Kishi[3], Takamasa Ueno[1], Andrew K. Sewell[1,2‡*], Chihiro Motozono[1‡*]

[1] Division of Infection and Immunity, Joint Research Center for Human Retrovirus infection, Kumamoto University, Kumamoto 8600811, Japan
[2] Division of Infection and Immunity, Cardiff University School of Medicine, CF14 4XN Cardiff, Wales, UK
[3] Department of Immunology, Faculty of Medicine, Academic Assembly, University of Toyama, Toyama 9300194, Japan
[4] Department of Applied Chemistry, Faculty of Science and Engineering, Kindai University, Osaka 577-8502, Japan
[5] Department of Immunology, Kochi University, Kochi 7838505, Japan
[6] Department of Immunology, Graduate School of Medical Sciences, Faculty of Life Sciences, Kumamoto University, Kumamoto 8608556, Japan
[7] Department of Pediatrics, Graduate School of Medical Sciences, Kumamoto University, Kumamoto 8608556, Japan
[8] Division of Infectious Diseases, Clinical Research Institute, NHO, Kyushu Medical Center, Fukuoka 8108563, Japan
[9] Internal Medicine, Clinical Research Institute, NHO, Kyushu Medical Center, Fukuoka 8108563, Japan
[10] Department of Hematology, Rheumatology and Infectious Diseases, Kumamoto University School of Medicine, Kumamoto University Hospital, Kumamoto 8608556, Japan

Prepared by Andrew Sewell
April 22nd, 2026

CONTENTS

Supplemental Tables S1-S6
Supplemental Figures S1-S8

Table S1: Donor information related to Fig. 1, 2 and 7.

Donor ID	Sex	Age	HLA-A24?	vaccinated?	Days after 2nd vax	Blood collection
**VKU-1**	Male	48	Positive	BNT162b2	35	03/05/2021
**VKU-2**	Male	37	Positive	BNT162b2	35	03/05/2021
**VKU-3**	Male	40	Positive	BNT162b2	35	03/05/2021
**VKU-5**	Male	34	Negative	BNT162b2	35	03/05/2021
**VKU-7**	Male	55	Positive	BNT162b2	31	08/05/2021
**VKU-8**	Male	55	Negative	BNT162b2	35	08/05/2021
**VKU-10**	Female	60	Positive	BNT162b2	48	03/05/2021
**VKU-11**	Female	39	Positive	BNT162b2	34	25/05/2021
**VKU-16**	Male	54	Negative	BNT162b2	35	08/05/2021
**VKU-17**	Male	35	Negative	BNT162b2	35	25/05/2021
**VKU-18**	Female	38	Positive	BNT162b2	35	25/05/2021
**VKU-19**	Female	36	Positive	BNT162b2	35	27/05/2021
**VKU-20**	Male	33	Negative	BNT162b2	34	25/05/2021
**VKU-21**	Female	27	Negative	BNT162b2	34	25/05/2021
**VKU-22**	Male	34	Positive	BNT162b2	36	27/05/2021
**VKU-23**	Male	54	Positive	BNT162b2	49	27/05/2021
**VKU-25**	Male	46	Negative	BNT162b2	34	25/05/2021
**VKU-27**	Male	43	Negative	BNT162b2	35	27/05/2021
**VKU-28**	Male	30	Positive	BNT162b2	35	10/06/2021
**VKU-41**	Male	34	Negative	BNT162b2	35	27/05/2021
**VKU-42**	Male	34	Positive	BNT162b2	35	27/05/2021
**VKU-43**	Male	38	Negative	BNT162b2	35	27/05/2021
**VKU-44**	Male	29	Positive	BNT162b2	31	25/05/2021
**VKU-45**	Male	27	Negative	BNT162b2	35	27/05/2021
**VKU-46**	Female	40	Negative	BNT162b2	35	27/05/2021
**VKU-47**	Female	57	Negative	BNT162b2	31	25/05/2021
**VKU-48**	Male	59	Positive	BNT162b2	35	27/05/2021
**GV9**	Female	24	Positive	BNT162b2	202	27/12/2021
**GV12**	Female	28	Negative	BNT162b2	23	01/07/2021
**GV15**	Female	23	Positive	BNT162b2	182	06/01/2022
**GV16**	Male	22	Positive	No	NA	17/05/2021
**GV16-1**	Male	22	Positive	BNT162b2	202	27/12/2021
**GV17**	Male	24	Negative	BNT162b2	21	29/06/2021
**GV19**	Male	24	Positive	BNT162b2	202	27/12/2021
**GV24**	Male	23	Positive	BNT162b2	212	06/01/2022
**GV25**	Male	24	Negative	BNT162b2	22	30/06/2021
**GV26**	Male	23	Positive	BNT162b2	212	06/01/2022
**GV27**	Female	23	Negative	BNT162b2	21	29/06/2021
**GV32**	Male	56	Positive	No	NA	11/05/2021
**GV32-1**	Male	56	Positive	BNT162b2	27	05/07/2021
**GV32-2**	Male	56	Positive	BNT162b2	195	20/12/2021
**GV33**	Male	39	Positive	No	NA	11/05/2021
**GV33-1**	Male	39	Positive	BNT162b2	24	28/07/2021
**GV33-2**	Male	39	Positive	BNT162b2	192	20/12/2021
**GV34**	Female	38	Positive	BNT162b2	24	05/07/2021
**GV35**	Male	52	Positive	BNT162b2	24	05/07/2021
**GV36**	Male	41	Positive	BNT162b2	21	05/07/2021
**GV36-1**	Male	41	Positive	BNT162b2	192	20/12/2021
**GV52**	Female	67	Positive	BNT162b2	21	05/07/2021
**GV59**	Male	37	Positive	BNT162b2	25	15/09/2021
**GV59-1**	Male	37	Positive	BNT162b2	126	20/12/2021
**GV60**	Male	51	Positive	mRNA-1273	116	20/12/2021

Table S2: Convalescent donor information related to Fig. 2 and 6.

Cohort	Donor ID	Sex	Age	HLA-A24?	vaccinated?	COVID-19 severity	Days post PCR+ or onset	Blood collection
**A24+** COVID-19 convalescents	**KK-008**	Male	63	Positive	No	Mild	17	19/08/2021
	**GV-38**	Male	23	Positive	No	Mild	18	20/05/2021
	**GV-41**	Male	33	Positive	No	Mild	16	20/05/2021
	**GV-42**	Male	25	Positive	No	Mild	32	03/06/2021
	**AK-16**	Female	46	Positive	No	Moderate	11	27/08/2021
	**AK-18**	Female	25	Positive	No	Moderate	10	03/09/2021
	**AK-20**	Female	58	Positive	No	Severe	NA	03/09/2021
	**AK-24**	Female	28	Positive	No	Severe	13	08/09/2021
	**AK-25**	Female	42	Positive	No	Severe	7	08/09/2021
	**IK-25**	Male	61	Positive	NA	Moderate	7	09/03/2022
	**IK-26**	Female	40	Positive	NA	Moderate	17	06/08/2021
	**IK-32**	Male	36	Positive	NA	Moderate	19	01/09/2021
**A24-** COVID-19 convalescents	**AK-12**	Male	53	Negative	No	Moderate	11	25/08/2021
	**AK-19**	Male	47	Negative	No	Severe	11	03/09/2021
	**AK-32**	Male	57	Negative	No	Moderate	10	13/09/2021
	**IK-21**	Female	71	Negative	NA	Moderate	16	30/07/2021
	**IK-22**	Male	33	Negative	NA	Mild	14	30/07/2021
	**IK-24**	Male	43	Negative	NA	Moderate	17	06/08/2021
	**IK-27**	Female	56	Negative	NA	Moderate	13	06/08/2021
	**IK-33**	Male	41	Negative	NA	Mild	34	06/09/2021
	**IK-34**	Male	38	Negative	NA	Mild	46	16/09/2021

NA – Not applicable

Table S3: HLA binding of NF9-5X peptides.

Name	Sequence	Normalized log Kd
NF9 (WT)	NYNY**L**YRLF	-6.83
NF9-5A	----**A**----	-6.90
NF9-5C	----**C**----	-6.12
NF9-5D	----**D**----	-6.17
NF9-5E	----**E**----	-5.90
NF9-5F	----**F**----	-6.72
NF9-5G	----**G**----	-6.56
NF9-5H	----**H**----	-6.93
NF9-5I	----**I**----	-7.03
NF9-5K	----**K**----	-6.94
NF9-5M	----**M**----	-6.85
NF9-5N	----**N**----	-7.00
NF9-5P	----**P**----	-6.82
NF9-5Q	----**Q**----	-6.55
NF9-5R	----**R**----	-6.35
NF9-5V	----**V**----	-6.68
NF9-5S	----**S**----	-6.62
NF9-5T	----**T**----	-6.71
NF9-5V	----**V**----	-6.68
NF9-5W	----**W**----	-6.14
NF9-5Y	----**Y**----	-6.50

Colors of text in name column match those used elsewhere in this study

Table S4: Crystallography statistics for X-ray crystallography structures in this study

PDB Entry	28IL	8RJH	8RJI
**Protein**	**P1-15:HLA A*2402-NF9**	**HLA A*2402-NF9_6F**	**HLA A*2402-NF9_5R**
**Data Collection**			
Diamond Beamline	I04	I04	I04
Date	28-04-2022	28-04-2022	28-04-2022
Wavelength	0.9795	0.9795	0.9795
**Crystal Data (outer shell statistics in brackets)**			
Crystallisation Conditions	0.1 M di-Sodium malonate, 0.1 M HEPES, 30% w/v Poly(acrylic acid sodium salt) 2,100, pH7	0.1M Sodium cacodylate, 20% PEG 4000, 15% Glycerol, pH 6	0.1M MES, 20% PEG 4000, 15% Glycerol, pH 7
a,b,c (Å)	200.44, 200.44, 156.27	344.8, 84.34, 91.45	103.28, 77.02, 111.82
α,β,γ (°)	90.0, 90.0, 120.0	90.00, 102.14, 90.00	90.00, 110.58, 90.00
Space group	P 3_1 2 1	C 1 2 1	P 1 2_1 1
Resolution (Å)	3.1 – 60.49	2.60 – 86.92	2.3 – 54.8
Outer shell	3.10 – 3.18	2.60 – 2.65	2.3 – 2.35
R-merge (%)	0.287 (3.769)	0.166 (1.581)	0.144 (1.088)
R-pim	0.091 (1.171)	0.101 (1.021)	0.089 (0.686)
R-meas (%)	0.300 (3.941)	0.195 (1.887)	0.170 (1.289)
CC1/2	0.997 (0.407)	0.995 (0.465)	0.996 (0.677)
I / σ(I)	8.8 (0.9)	7.7 (1.0)	8.0 (1.5)
Completeness (%)	100 (100)	100 (99.5)	99.6 (96.3)
Multiplicity	21 (22)	7.1 (6.6)	7.0 (6.9)
Total Measurements	1,387,790 (101,844)	564,978 (29,665)	512,213 (29,794)
Unique Reflections	65,958 (4,631)	79,258 (4,490)	73,135 (4,344)
Wilson B-factor(Å^2)	93	51.1	33.7
**Refinement Statistics**			
Non-H Atoms	14,898	19,159	13,033
R-work reflections	62481	75,257	73,254
R-free reflections	3,218	3,967	3,602
R-work/R-free (%)	19.0 / 23.1	22.4 / 26.4	21.7 / 26.5
**rms deviations (ML target in brackets)**			
Bond lengths (Å)	0.006 (0.012)	0.010 (0.013)	0.012 (0.013)
Bond Angles (°)	1.504 (1.772)	1.372 (1.648)	1.447 (1.647)
[1]Coordinate error	0.355	0.323	0.4401
Mean B value (Å^2)	112.3	63.2	39.2
**Ramachandran Statistics**			
Favoured/Outliers	1459 / 20	2152 / 1	1429 / 6
%	89 / 1	95 / 0	94 / 0

* One crystal was used for determining each structure.

[1] Coordinate Estimated Standard Uncertainty in (Å), calculated based on maximum likelihood statistics.

Table S5. Molecular contacts between the P1-15 T-cell Receptor and the A24/NF9 peptide:MHC complex. Note that the P1-15 TCR β-chain construct contained a short N-terminal extension resulting from the expression system, and the PDB numbering therefore begins from the first residue of this construct. For consistency with the published literature (e.g., PDB: 8YE4), amino acid numbering in this manuscript follows the canonical TCR sequence i.e., -3 relative to the PDB files for the β chain.

CDR loop	TCR residue	Peptide residue	MHC residue	VdWs (≤4 Å)	H-bonds (≤3.4 Å)
CDR1α	Ala29	Tyr4		3	
	Gln31		Gln155	2	
		Tyr4		8	
		Tyr6		1	
	Ser32	Tyr6		2	
CDR2α	Tyr51		Ala150	1	
			His151	23	
			Glu154	1	
	Ser52		Glu154	2	
	Ser53		Glu154	4	
FWα	Arg66		Ala158	3	
CDR3α	Asn91	Tyr6		4	
	Leu93		Glu62	1	
			Gly65	2	
			Lys66	3	
	Asn95		Gly65	1	
	Ser96		Gly65	1	
			Gly68	1	
	Tyr98		Lys66	2	
			Ala69	1	
		Asn3		1	
		Tyr4		8	
		Leu5		5	1
		Tyr6		4	
CDR1β	Asn30		Glu76		1
CDR3β	Ser96	Leu8		2	
	Gly97	Tyr6		1	
	Gly98	Tyr6		2	
	Tyr99	Tyr6		1	
		Arg7		12	1
		Leu8		5	

Table S1: Donor information related to Fig. 1, 2 and 7.

Donor ID	Sex	Age	HLA-A24?	vaccinated?	Days after 2nd vax	Blood collection
VKU-1	Male	48	Positive	BNT162b2	35	03/05/2021
VKU-2	Male	37	Positive	BNT162b2	35	03/05/2021
VKU-3	Male	40	Positive	BNT162b2	35	03/05/2021
VKU-5	Male	34	Negative	BNT162b2	35	03/05/2021
VKU-7	Male	55	Positive	BNT162b2	31	08/05/2021
VKU-8	Male	55	Negative	BNT162b2	35	08/05/2021
VKU-10	Female	60	Positive	BNT162b2	48	03/05/2021
VKU-11	Female	39	Positive	BNT162b2	34	25/05/2021
VKU-16	Male	54	Negative	BNT162b2	35	08/05/2021
VKU-17	Male	35	Negative	BNT162b2	35	25/05/2021
VKU-18	Female	38	Positive	BNT162b2	35	25/05/2021
VKU-19	Female	36	Positive	BNT162b2	35	27/05/2021
VKU-20	Male	33	Negative	BNT162b2	34	25/05/2021
VKU-21	Female	27	Negative	BNT162b2	34	25/05/2021
VKU-22	Male	34	Positive	BNT162b2	36	27/05/2021
VKU-23	Male	54	Positive	BNT162b2	49	27/05/2021
VKU-25	Male	46	Negative	BNT162b2	34	25/05/2021
VKU-27	Male	43	Negative	BNT162b2	35	27/05/2021
VKU-28	Male	30	Positive	BNT162b2	35	10/06/2021
VKU-41	Male	34	Negative	BNT162b2	35	27/05/2021
VKU-42	Male	34	Positive	BNT162b2	35	27/05/2021
VKU-43	Male	38	Negative	BNT162b2	35	27/05/2021
VKU-44	Male	29	Positive	BNT162b2	31	25/05/2021
VKU-45	Male	27	Negative	BNT162b2	35	27/05/2021
VKU-46	Female	40	Negative	BNT162b2	35	27/05/2021
VKU-47	Female	57	Negative	BNT162b2	31	25/05/2021
VKU-48	Male	59	Positive	BNT162b2	35	27/05/2021
GV9	Female	24	Positive	BNT162b2	202	27/12/2021
GV12	Female	28	Negative	BNT162b2	23	01/07/2021
GV15	Female	23	Positive	BNT162b2	182	06/01/2022
GV16	Male	22	Positive	No	NA	17/05/2021
GV16-1	Male	22	Positive	BNT162b2	202	27/12/2021
GV17	Male	24	Negative	BNT162b2	21	29/06/2021
GV19	Male	24	Positive	BNT162b2	202	27/12/2021
GV24	Male	23	Positive	BNT162b2	212	06/01/2022
GV25	Male	24	Negative	BNT162b2	22	30/06/2021
GV26	Male	23	Positive	BNT162b2	212	06/01/2022
GV27	Female	23	Negative	BNT162b2	21	29/06/2021
GV32	Male	56	Positive	No	NA	11/05/2021
GV32-1	Male	56	Positive	BNT162b2	27	05/07/2021
GV32-2	Male	56	Positive	BNT162b2	195	20/12/2021
GV33	Male	39	Positive	No	NA	11/05/2021
GV33-1	Male	39	Positive	BNT162b2	24	28/07/2021
GV33-2	Male	39	Positive	BNT162b2	192	20/12/2021
GV34	Female	38	Positive	BNT162b2	24	05/07/2021
GV35	Male	52	Positive	BNT162b2	24	05/07/2021
GV36	Male	41	Positive	BNT162b2	21	05/07/2021
GV36-1	Male	41	Positive	BNT162b2	192	20/12/2021
GV52	Female	67	Positive	BNT162b2	21	05/07/2021
GV59	Male	37	Positive	BNT162b2	25	15/09/2021
GV59-1	Male	37	Positive	BNT162b2	126	20/12/2021
GV60	Male	51	Positive	mRNA-1273	116	20/12/2021

Table S2: Convalescent donor information related to Fig. 2 and 6.

Cohort	Donor ID	Sex	Age	HLA-A24?	vaccinated?	COVID-19 severity	Days post PCR+ or onset	Blood collection
**A24⁺** COVID-19 convalescents	**KK-008**	Male	63	Positive	No	Mild	17	19/08/2021
	**GV-38**	Male	23	Positive	No	Mild	18	20/05/2021
	**GV-41**	Male	33	Positive	No	Mild	16	20/05/2021
	**GV-42**	Male	25	Positive	No	Mild	32	03/06/2021
	**AK-16**	Female	46	Positive	No	Moderate	11	27/08/2021
	**AK-18**	Female	25	Positive	No	Moderate	10	03/09/2021
	**AK-20**	Female	58	Positive	No	Severe	NA	03/09/2021
	**AK-24**	Female	28	Positive	No	Severe	13	08/09/2021
	**AK-25**	Female	42	Positive	No	Severe	7	08/09/2021
	**IK-25**	Male	61	Positive	NA	Moderate	7	09/03/2022
	**IK-26**	Female	40	Positive	NA	Moderate	17	06/08/2021
	**IK-32**	Male	36	Positive	NA	Moderate	19	01/09/2021
**A24⁻** COVID-19 convalescents	**AK-12**	Male	53	Negative	No	Moderate	11	25/08/2021
	**AK-19**	Male	47	Negative	No	Severe	11	03/09/2021
	**AK-32**	Male	57	Negative	No	Moderate	10	13/09/2021
	**IK-21**	Female	71	Negative	NA	Moderate	16	30/07/2021
	**IK-22**	Male	33	Negative	NA	Mild	14	30/07/2021
	**IK-24**	Male	43	Negative	NA	Moderate	17	06/08/2021
	**IK-27**	Female	56	Negative	NA	Moderate	13	06/08/2021
	**IK-33**	Male	41	Negative	NA	Mild	34	06/09/2021
	**IK-34**	Male	38	Negative	NA	Mild	46	16/09/2021

NA – Not applicable

Table S3: HLA binding of NF9-5X peptides.

Name	Sequence	Normalized log Kd
NF9 (WT)	NYNY**L**YRLF	-6.83
NF9-5A	----**A**----	-6.90
NF9-5C	----**C**----	-6.12
NF9-5D	----**D**----	-6.17
NF9-5E	----**E**----	-5.90
NF9-5F	----**F**----	-6.72
NF9-5G	----**G**----	-6.56
NF9-5H	----**H**----	-6.93
NF9-5I	----**I**----	-7.03
NF9-5K	----**K**----	-6.94
NF9-5M	----**M**----	-6.85
NF9-5N	----**N**----	-7.00
NF9-5P	----**P**----	-6.82
NF9-5Q	----**Q**----	-6.55
NF9-5R	----**R**----	-6.35
NF9-5V	----**V**----	-6.68
NF9-5S	----**S**----	-6.62
NF9-5T	----**T**----	-6.71
NF9-5V	----**V**----	-6.68
NF9-5W	----**W**----	-6.14
NF9-5Y	----**Y**----	-6.50

Colors of text in name column match those used elsewhere in this study

Table S4: Crystallography statistics for X-ray crystallography structures in this study

**PDB Entry**	**28IL**	**8RJH**	**8RJI**
**Protein**	**P1-15:HLA A*2402-NF9**	**HLA A*2402-NF9_6F**	**HLA A*2402-NF9_5R**
**Data Collection**			
Diamond Beamline	I04	I04	I04
Date	28-04-2022	28-04-2022	28-04-2022
Wavelength	0.9795	0.9795	0.9795
**Crystal Data (outer shell statistics in brackets)**			
Crystallisation Conditions	0.1 M di-Sodium malonate, 0.1 M HEPES, 30% w/v Poly(acrylic acid sodium salt) 2,100, pH7	0.1M Sodium cacodylate, 20% PEG 4000, 15% Glycerol, pH 6	0.1M MES, 20% PEG 4000, 15% Glycerol, pH 7
a,b,c (Å)	200.44, 200.44, 156.27	344.8, 84.34, 91.45	103.28, 77.02, 111.82
α,β,γ (°)	90.0, 90.0, 120.0	90.00, 102.14, 90.00	90.00, 110.58, 90.00
Space group	P 3_1 2 1	C 1 2 1	P 1 2_1 1
Resolution (Å)	3.1 – 60.49	2.60 – 86.92	2.3 – 54.8
Outer shell	3.10 – 3.18	2.60 – 2.65	2.3 – 2.35
R-merge (%)	0.287 (3.769)	0.166 (1.581)	0.144 (1.088)
R-pim	0.091 (1.171)	0.101 (1.021)	0.089 (0.686)
R-meas (%)	0.300 (3.941)	0.195 (1.887)	0.170 (1.289)
CC1/2	0.997 (0.407)	0.995 (0.465)	0.996 (0.677)
I / σ(I)	8.8 (0.9)	7.7 (1.0)	8.0 (1.5)
Completeness (%)	100 (100)	100 (99.5)	99.6 (96.3)
Multiplicity	21 (22)	7.1 (6.6)	7.0 (6.9)
Total Measurements	1,387,790 (101,844)	564,978 (29,665)	512,213 (29,794)
Unique Reflections	65,958 (4,631)	79,258 (4,490)	73,135 (4,344)
Wilson B-factor($Å^2$)	93	51.1	33.7
**Refinement Statistics**			
Non-H Atoms	14,898	19,159	13,033
R-work reflections	62481	75,257	73,254
R-free reflections	3,218	3,967	3,602
R-work/R-free (%)	19.0 / 23.1	22.4 / 26.4	21.7 / 26.5
**rms deviations (ML target in brackets)**			
Bond lengths (Å)	0.006 (0.012)	0.010 (0.013)	0.012 (0.013)
Bond Angles (°)	1.504 (1.772)	1.372 (1.648)	1.447 (1.647)
[1]Coordinate error	0.355	0.323	0.4401
Mean B value ($Å^2$)	112.3	63.2	39.2
**Ramachandran Statistics**			
Favoured/Outliers	1459 / 20	2152 / 1	1429 / 6
%	89 / 1	95 / 0	94 / 0

* One crystal was used for determining each structure.

[1] Coordinate Estimated Standard Uncertainty in (Å), calculated based on maximum likelihood statistics.

Table S5. Molecular contacts between the P1-15 T-cell Receptor and the A24/NF9 peptide:MHC complex. Note that the P1-15 TCR β-chain construct contained a short N-terminal extension resulting from the expression system, and the PDB numbering therefore begins from the first residue of this construct. For consistency with the published literature (e.g., PDB: 8YE4), amino acid numbering in this manuscript follows the canonical TCR sequence i.e., -3 relative to the PDB files for the β chain.

CDR loop	TCR residue	Peptide residue	MHC residue	VdWs (≤4 Å)	H-bonds (≤3.4 Å)
CDR1α	Ala29	Tyr4		3	
	Gln31		Gln155	2	
		Tyr4		8	
		Tyr6		1	
	Ser32	Tyr6		2	
CDR2α	Tyr51		Ala150	1	
			His151	23	
			Glu154	1	
	Ser52		Glu154	2	
	Ser53		Glu154	4	
FWα	Arg66		Ala158	3	
CDR3α	Asn91	Tyr6		4	
	Leu93		Glu62	1	
			Gly65	2	
			Lys66	3	
	Asn95		Gly65	1	
	Ser96		Gly65	1	
			Gly68	1	
	Tyr98		Lys66	2	
			Ala69	1	
		Asn3		1	
		Tyr4		8	
		Leu5		5	1
		Tyr6		4	
CDR1β	Asn30		Glu76		1
CDR3β	Ser96	Leu8		2	
	Gly97	Tyr6		1	
	Gly98	Tyr6		2	
	Tyr99	Tyr6		1	
		Arg7		12	1
		Leu8		5	

Supplemental Table 6. Cell identities in Figure 4

Cluster ID	Top GSEA term	RSV⁺	Original identity	ISGs	Name
0	HALLMARK_E2F_TARGET	neg	iAT2s (mono- and coculture)	neg	Proliferating iAT2s (S)
1	HALLMARK_ESTROGEN_ RESPONSE_ EARLY	neg	iAT2s (mono- and coculture)	pos	Antiviral bystander iAT2s
2	HALLMARK_UV_RESPONSE_DN	neg	iAT2s (co)	pos	Antiviral bystander iAT2s (cocultured)
3	HALLMARK_INTERFERON_ GAMMA_ RESPONSE	pos	iMacs (mono)	pos	RSV⁺ iMacs (monoculture)
4	HALLMARK_E2F_TARGET	neg	iAT2s (mono- and coculture)	pos/neg	Proliferating iAT2s (G2M)
5	HALLMARK_OXIDATIVE_ PHOSPHORYLATION	neg	iAT2s (mono- and coculture)	neg	iAT2s
6	HALLMARK_MYC_ TARGETS_V1	neg	iAT2s (mono- and coculture)	neg	iAT2s
7	HALLMARK_E2F_TARGET	neg	iAT2s (co)	pos/ neg	Proliferating iAT2s (cocultured)
8	HALLMARK_TNFA_ SIGNALLING_VIA_ NFKB	neg	iMacs (mono)	pos	Antiviral bystander iMacs (monoculture)
9	HALLMARK_INTERFERON_ GAMMA_ RESPONSE	neg	iMacs (co)	pos	Antiviral bystander iMacs (coculture)
10	HALLMARK_INTERFERON_ GAMMA_ RESPONSE	pos	iMacs (co)	pos	RSV⁺ iMacs (coculture)
11	HALLMARK_OXIDATIVE_ PHOSPHORYLATION	neg	iAT2s (mono- and coculture)	neg	iAT2s
12	HALLMARK_HYPOXIA	pos	iAT2s (mono- and coculture)	pos	RSV⁺ iAT2s

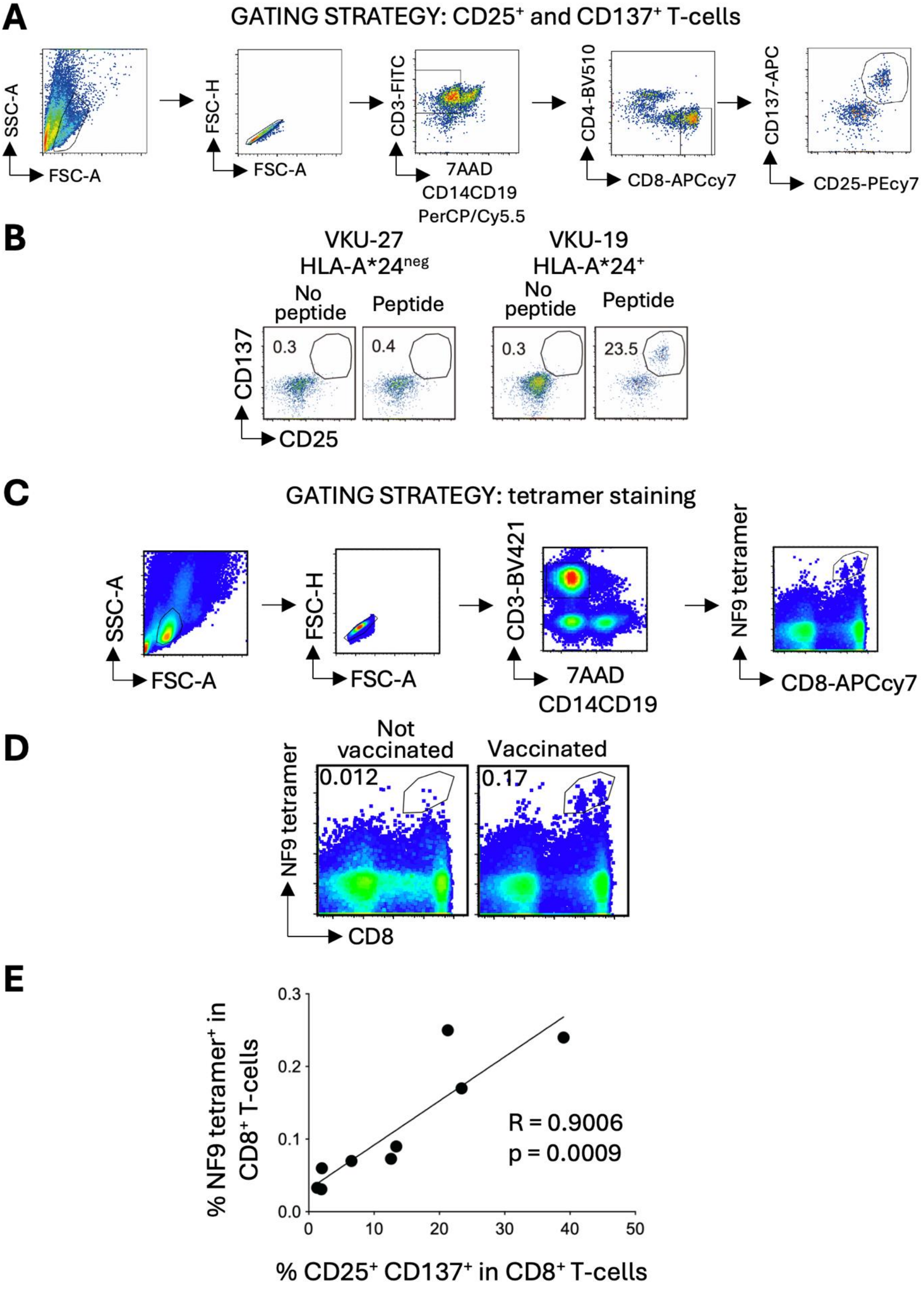

Supplemental Figure S1. Flow cytometry gating strategies, representative flow cytometry plots, and correlation between activation status and tetramer staining for the HLA-A*24:02 NF9 epitope. (**A**) Flow cytometry gating strategy of $CD25^{+}CD137^{+}$ T-cell lines from donor VKU19. (**B**) $CD8^{+}CD25^{+}CD137^{+}$ data for an HLA-A*24:02 positive and negative donor. (**C**) HLA-A*24:02 NF9 tetramer staining of PBMCs from donor VKU-19. (**E**) Correlation between the frequency of $CD25^{+}CD137^{+}CD8^{+}$ activated T cells and that of HLA-A*24:02 NF9 $tetramer^{+}CD8^{+}$ T cells in HLA-$A^{*}24{:}02^{+}$ vaccinated donors (n = 9). R = 0.9006 and *** p = 0.0009 by two-tailed Person test.

GV33

TRAV	TRAJ	CDR3α	TRBV	TRBD	TRBJ	CDR3β	%
12-1*01	41*01	CVVNLLNSNSGYALNF	6-1*01	2*01	2-7*01	CASSEAGGYEQYF	12.5
14/DV4*02	45*01	CAMREPPLIRGADGLTF	2*01	1*01	2-7*01	CASSEGAGGYEQYF	8.3
14/DV4*02	50*01	CAMRELGVKTSYDKVIF	6-1*01	2*02	2-7*01	CASSEGRGYEQYF	4.2
8-6*02	17*01	SPGAAGNKLTF	6-1*01	2*01	2-7*01	CASSETGGYEQYF	4.2
16*01	6*01	CALSGPSGAGGSYIPTF	5-4*01	2*01	2-7*01	CASSETSGYEQYF	4.2
12-2*01	20*01	CAVTHLARADYKLSF	5-1*01	1*01	2-7*01	CASSDRTGYEQYF	4.2
12-1*01	5*01	CVVKGTGRRALTF	11-1*01	1*01	2-7*01	CASSLWQGYEQFF	4.2
21*02	47*01	CAVNSYGNKLVF	5-1*01	1*01	2-7*01	CASSPGTGYEQYF	4.2
4*01	36*01	CLVGDGGGANNLFF	4-1*01	2*01	2-7*01	CASSQSLGYEQYF	4.2
12-2*02	35*01	CAVNGFGNVLHC	10-1*01	2*01	2-7*01	CASSESIAYEQYF	4.2
21*02	26*01	CAVPNNYGQNFVF	2*01	1*01	2-7*01	CASSEFGVGYEQYF	4.2
12-2*01	40*01	CAVNSGTYKYIF	5-1*01	1*01	2-7*01	CASSEGAGGYEQYF	4.2
12-1*01	8*01	CVVSTPLMNTGFQKLVF	6-1*01	1*01	2-7*01	CASSEGQGPYEQYF	4.2
26-2*01	32*02	CILFYGGATNKLIF	2*01	1*01	2-7*01	CASSEGQLPYEQYF	4.2
14/DV4*02	52*01	CAIVGGTSYGKLTF	19*01	1*01	2-7*01	CASSLAEGTEQYF	4.2
27*01	23*01	CAGARNQGGKLIF	27*01	1*01	1-1*01	CASSLGGHNPEEAFF	4.2
23/DV6*01	45*01	CAAGSGGGADGLTF	2*01	2*02	2-7*01	CASNFEGGSSYEQYF	4.2
17*01	20*01	CATAPWDYKLSF	5-6*01	1*01	1-1*01	CASSLGQGTEAFF	4.2
17*01	40*01	CATVTYKYIF	5-4*01	-	2-7*01	CASSLGSADEQYF	4.2
1-1*01	4*01	CAAASGGYNKLIF	27*01	1*01	2-1*01	CASSLSLGEQFF	4.2
12-1*01	9*01	CVVTLYTGGFKTIF	7-9*03	1*01	1-1*01	CASSPQGADEAFF	4.2

GV34

TRAV	TRAJ	CDR3α	TRBV	TRBD	TRBJ	CDR3β	%
13-1*02	44*01	SRNTGTASKLTF	5-1*01	1*01	2-7*01	CASSFGQGYEQYF	8.6
12-1*01	33*01	CVVNALMDSNYQLIW	5-1*01	1*01	2-7*01	CASSLGQGYEQYF	5.7
12-1*01	33*01	CVVNLFDSNYQLIW	2*01	1*01	2-7*01	CASSEGAGYEQYF	2.9
12-1*01	28*01	CVVNMLYSGAGSYQLTF	6-1*01	2*01	1-1*01	CASSESAGYEAFF	2.9
12-1*01	28*01	CVVNVPLGGAGSYQLTF	6-4*01	1*01	2-7*01	CASSEGQGYEQYF	2.9
12-1*01	41*01	CVVNGRRNSGYALNF	10-1*01	-	2-7*01	CASSESFGYEQYF	2.9
12-1*01	28*01	CVVNKLDSGAGSYQLTF	5-1*01	2*01	2-7*01	CASSLAGGYEQYF	2.9
12-1*01	8*01	CVVNDRWNTGFQKLVF	2*01	1*01	2-7*01	CASSEGQAYEQYF	2.9
12-1*01	8*01	CVAREGFQKLVF	9*01	2*01	2-7*01	CASSETPAYEQYF	2.9
5*01	32*01	CAPRGATTKLIF	6-1*01	1*01	2-7*01	CASSEFQGYEQYF	2.9
29/DV5*04	54*01	CAASVIQGAQKLVF	4-1*01	1*01	2-7*01	CASSPGRSYEQYF	2.9
20*04	49*01	CAVPGYSRATGNQLTF	2*01	1*01	2-7*01	CASMVGLTYEQYF	2.9
38-2/DV8*01	42*01	CAYRSAWWGPSQGNLIF	3-1*01	1*01	2-7*01	CASSWGQDAYEQYF	2.9
5*01	42*01	CAESGGSQGNLIF	5-1*01	1*01	2-7*01	CASMGQNYEQYF	2.9
21*01	58*01	CAVRPETSGSRLTF	6-5*01	2*01	2-1*01	CASSALGLAAYNEQFF	2.9
27*01	35*01	CAGAVGFGNVLHC	2*01	1*01	1-2*01	CASSEAGAVYGYTF	2.9
17*01	52*01	CATTNAGGTSYGKLTF	2*01	2*02	2-7*01	CASSEWVGSSYEQYF	2.9
8-6*02	18*01	CAVSDRGSTLGRLYF	28*01	1*01	1-1*01	CASSFGAYDTEAFF	2.9
12-2*01	54*01	CAVNTGAQKLVF	6-6*01	1*01	2-1*01	CASSFPTAGGARNEQFF	2.9
17*01	8*01	CATDRMNTGFQKLVF	19*01	1*01	2-7*01	CASSIGQTPTYEQYF	2.9
20*02	39*01	CAVQNNNAGNMLTF	5-1*01	1*01	1-1*01	CASSLESGTEAFF	2.9
12-3*01	57*01	CAMSIQGGSEKLVF	5-6*01	2*02	1-5*01	CASSLGSSAQHF	2.9
8-6*01	11*01	CAVRRYSTLTF	5-6*01	1*01	2-7*01	CASSLQGARPYEQYF	2.9
17*01	17*01	CALGGAAGNKLTF	13*01	-	2-3*01	CASSLTDTQYF	2.9
29/DV5*04	39*01	CAANDAGNMLTF	4-1*01	2*01	2-7*01	CASSQDPALAGPYEQYF	2.9
10*02	15*01	CVVSARLNTDAASNLTF	11-3*01	1*01	2-1*01	CASSSRARPNEQFF	2.9
30*05	34*01	CGTALPYNTDKLIF	7-2*02	2*01	2-5*01	CASSSRTDLKETQYF	2.9
5*01	44*01	CAETKKNTGTASKLTF	7-6*01	1*01	2-5*01	CASSSSRSGITQYF	2.9
19*01	36*01	CALSESGANNLFF	7-9*01	1*01	2-5*01	CASSSWRQQETQYF	2.9
5*01	30*01	CVVMNRDDKIIF	6-2*01	2*01	2-1*01	CASSYSAGEQFF	2.9
12-1*01	12*01	CVVITMDSSYKLIF	20-1*01	1*01	2-7*01	CSARPRQGVYEQYF	2.9
12-2*01	54*01	CAVNEIQGAQKLVF	29-1*01	1*01	2-5*01	CSVTPGGETQYF	2.9

VK22

TRAV	TRAJ	CDR3α	TRBV	TRBD	TRBJ	CDR3β	%
4*01	3*01	CLVGGSYSSASKIIF	2*01	1*01	2-7*01	CASSEGRGYEQYF	13.0
12-1*01	33*01	CVVNVYMDSNYQLIW	2*01	2*01	2-7*01	CASSESAGYEQYF	4.3
12-1*01	28*01	CVVNMLGSGAGSYQLTF	5-4*01	2*02	2-7*01	CASSLAGGYEQYF	4.3
12-1*01	33*01	CVVNGDSNYQLIW	4-1*01	1*01	2-7*01	CASSQQHGYEQYF	4.3
12-1*01	42*01	CVVTGGSQGNLIF	20-1*02	2*01	2-7*01	CSARDWLGTSDYEQYF	4.3
6*03	47*01	CALAEYGNKLVF	6-5*01	-	2-7*01	CASSYSFSYEQYF	4.3
29/DV5*04	37*01	CAAPGTSGNTGKLIF	15*02	1*01	2-7*01	CATSRILGYEQYF	4.3
5*01	20*01	CAETPSRGLSF	4-1*01	2*01	2-7*01	CASSQDAGEQYF	4.3
2*01	11*01	CAVEGYSGYSTLTF	12-4*01	2*01	1-1*01	CASLSGEAFF	4.3
20*02	42*01	CALQTNYGGSQGNLIF	9*01	2*02	2-1*01	CASSAGLEGGGTYNEQFF	4.3
24*01	32*02	CASYGGATNKLIF	2*01	2*01	2-7*01	CASSEFRGATYEQYF	4.3
38-2/DV8*01	43*01	CALNQNNNDMRF	12-3*01	2*02	2-3*01	CASSLTAGGPRTRTQYF	4.3
12-2*02	9*01	CAVRGFKTIF	12-3*01	1*01	2-1*01	CASSPAPGSGGNEQFF	4.3
12-2*02	20*01	CAVTNDYKLSF	6-5*01	2*01	2-3*01	CASSPHLGGGEDTQYF	4.3
26-1*01	23*01	CIVDPWGGGKLIF	9*01	1*01	1-1*01	CASSSSRQGNTEAFF	4.3
20*02	18*01	CAVQVWDRGSTLGRLYF	30*01	1*01	1-1*01	CAWSIQPGGTEAFF	4.3
3*01	6*01	CAVRDIRGSYIPTF	30*01	1*01	1-1*01	CAWSVQNTEAFF	4.3
13-1*02	20*01	CAASLAGNDYKLSF	20-1*05	2*01	2-7*01	CSARDRVRIYEQYF	4.3
4*01	5*01	CLVYRRALTF	29-1*01	1*01	1-1*01	CSVGDGNTEAFF	4.3
12-2*02	49*01	CAVNTPITNTGNQFYF	29-1*01	-	2-7*01	CSVTRSYEQYF	4.3
41*01	58*01	CAVPTSGYRLTF	9*03	2*02	2-5*01	CTSSKPAGGNTQYF	4.3

VK48

TRAV	TRAJ	CDR3α	TRBV	TRBD	TRBJ	CDR3β	%
12-1*01	28*01	CVVNRLQSGAGSYQLTF	2*01	2*01	2-7*01	CASSEAGGYEQYF	11.1
4*01	9*01	CLVGDIRHTGGFKTIF	2*01	-	1-6*02	CASSEDSPLHF	11.1
12-1*01	53*01	CVVNTLNSGGSNYKLTF	4-2*01	1*01	2-7*01	CASSPTGGYEQYF	5.6
12-1*01	12*01	CVVTVPPAMDSSYKLIF	6-1*01	1*01	2-7*01	CASSRKDGYEQYF	5.6
12-1*01	41*01	CVVNMLAGNSGYALNF	2*01	1*01	2-7*01	CASSDQQGYEQYF	5.6
12-1*01	33*01	CVVNILRDSNYQLIW	25-1*01	1*01	2-7*01	CASSEGQGYEQYF	5.6
12-1*01	20*01	CVVNCLREDDYKLSF	6-1*01	2*02	2-7*01	CASSEGRGYEQYF	5.6
16*01	4*01	CALSLFSGGYNKLIF	4-1*01	1*01	2-7*01	CASSQGQGYEQYF	5.6
23/DV6*02	49*01	CPAGGNQFYF	6-1*01	1*01	2-7*01	CASSEWVGYEQYF	5.6
17*01	20*01	CATDTNDYKLSF	19*01	1*01	1-2*01	CASSMRGQGYGYTF	5.6
20*02	4*01	CAVQPSFSGGYNKLIF	2*01	1*01	2-7*01	CASSEGQGPYEQYF	5.6
27*01	37*01	RAGAGGGTGELTF	6-1*01	1*01	1-5*01	CASSEWGIGDNQPQHF	5.6
27*01	42*01	CAASMGGSQGNLIF	11-2*01	1*01	2-4*01	CASSLGPVLAKNIQYF	5.6
14/DV4*02	29*01	CAMRPNSGNTPLVF	4-1*01	1*01	2-7*01	CASSQGRQGAAYEQYF	5.6
8-6*02	37*02	CAVSDRASNTGKLIF	4-1*01	1*01	1-1*01	CASSQVTGGWTEAFF	5.6
23/DV6*01	28*01	CAASTPGAGSYQLTF	6-5*01	1*01	2-7*01	CASSSSVQDIVEF	5.6
12-3*01	54*01	CAMRAPGAQKLVF	9*02	2*01	2-7*01	CASSVGPGLAAYEQYF	5.6

GV36

TRAV	TRAJ	CDR3α	TRBV	TRBD	TRBJ	CDR3β	%
1-2*01	23*01	CAVRDGSTQGGKLIF	13*01	1*01	2-1*01	CASSFPRNNEQFF	10.0
12-1*01	26*01	CVVNGRNYGQNFVF	4-1*01	2*01	2-7*01	CASSQPGGYEQSF	5.0
12-2*02	33*01	CAVNGLKDSNYQLIW	6-1*01	2*01	2-7*01	CASSEGGGYEQYF	5.0
17*01	37*02	CATHGSSNTGKLIF	7-3*01	-	2-7*01	CASSLFLAYEQYF	5.0
25*01	44*01	CAGKTGTASKLTF	27*01	2*02	2-7*01	CASSLGRLYEQYF	5.0
19*01	49*01	CALWCGNQFYF	27*01	1*01	2-1*01	CASGSNEQFF	5.0
3*01	3*01	CAVRDDYSSASKIIF	19*01	1*01	2-3*01	CASRDREDTQYF	5.0
10*01	43*01	CVVNPYNNNDMRF	9*01	2*02	2-7*01	CASSAGLAGAYEQYF	5.0
5*01	31*01	CAETSHNNARLMF	2*01	1*01	2-3*01	CASSESTGTDTQYF	5.0
13-1*02	11*01	CAPMNSGYSTLTF	7-9*01	1*01	2-6*01	CASSHLWVSGANVLTF	5.0
24*01	36*01	CARPGFRQTGANNLFF	3-1*01	1*01	2-1*01	CASSQDMDPYNEQFF	5.0
10*01	41*01	VVSGWSGYALIF	6-2*01	1*01	2-7*01	CASSYETGSSYEQYF	5.0
41*01	45*01	CASPGNSGGGADGLTF	25-1*01	1*01	2-6*01	CASTEGDVLTF	5.0
1-2*01	30*01	CAPTSDDKIIF	15*02	1*01	1-2*01	CATSPPGSGGYTF	5.0
12-2*02	35*01	CAVNHGLGGFGNVLHC	24-1*01	2*01	1-1*01	CATSVSGNTEAFF	5.0
14/DV4*02	33*01	CAMREGPMDSNYQLIW	7-9*01	1*01	1-6*01	CAVQGSYNSPLHF	5.0
3*01	37*01	VRCESQGGRSTGQLIF	30*01	1*01	2-5*01	CAWSVGRNGGETQYF	5.0
35*03	49*01	CAGQHGNQFYF	29-1*01	2*02	2-1*01	CSVEGTSGRSYNEQFF	5.0
8-2*03	8*01	CVVSDKGFQKLVF	2*01	2*02	2-2*01	VPSALLAGGPGSCF	5.0

VKU7

TRAV	TRAJ	CDR3α	TRBV	TRBD	TRBJ	CDR3β	%
13-2*01	11*01	CAEIALMNSGYSTLTF	2*01	1*01	2-7*01	CASSENRGYEQYF	4.0
12-1*02	33*01	CVVYGILDGNYQFTW	4-1*01	1*01	2-7*01	CASSRTGGYEQYF	4.0
2*01 F	37*01	CAAPLHSGNTGKLIF	6-6*01	1*01	2-7*01	CASSVGQGYEQYF	4.0
12-1*01	12*01	CVVNIMDSSYKLIF	2*01	1*01	2-7*01	CASSVSQGYEQYF	4.0
12-1*01	33*01	CVVNALRDSNYQLIW	6-1*01	1*01	2-7*01	CASSGGQGYEQYF	4.0
21*02	4*01	CGACHMFSGGYNKLIF	5-4*01	1*01	2-7*01	CASSLGVTYEQYF	4.0
6*02	49*01	CVIIPGTQFYF	2*01	1*01	2-7*01	CASRGQGGNEQYF	4.0
8-3*01	48*01	CAVRFNFGNEKLTF	9*01	1*01	1-1*01	CASSAPAGTEAFF	4.0
8-1*01	22*01	CLLLLRCGTRQLTF	6-6*01	1*01	2-5*01	CASSDATTQETQYF	4.0
8-4*01	31*01	CAVSETVNNARLMF	25-1*01	2*01	2-5*01	CASSEWGGQETQYF	4.0
3*01	15*01	CAVRERDQAGTALIF	9*01	2*01	2-5*01	CASSGTGGQETQYF	4.0
3*01	3*01	CAVRDPGYSSASKIIF	19*01	2*01	2-3*01	CASSIDLGTDTQYF	4.0
14/DV4*02	53*01	CAMRPNSGGSNYKLTF	11-3*01	2*01	2-7*01	CASSLGVPGLSYEQYF	4.0
8-1*01	37*01	CAVNSGNTGKGIF	11-2*01	2*01	2-7*01	CASSLPGGQDYEQYF	4.0
17*01	23*01	CATDDNQGGKLIF	12-3*01	-	2-7*01	CASSLSYEQYF	4.0
3*01	29*01	CAVRDICGNTPLVF	7-9*03	2*02	2-7*01	CASSLTGSPYEQYF	4.0
8-4*01	44*01	CAVRLYTGTASKLTF	14*02	2*02	1-4*01	CASSQGEVRNEKLFF	4.0
8-4*01	43*01	CAVSLNNNDMRF	5-4*01	1*01	2-7*01	CASSRTGQVNYEQYF	4.0
30*01	24*02	CGTTDSWGKLQF	7-3*01	1*01	2-3*01	CASSSGPADTQYF	4.0
9-2*01	52*01	CALSDNAGGTSYGKLTF	6-6*01	1*01	1-1*01	CASSYGSPNTEAFF	4.0
13-1*01	54*01	CAANPTQKLVF	7-9*03	2*02	2-1*01	CASSYPTSGSSYNEQFF	4.0
14/DV4*02	52*01	CAMRAGTSYGKLTF	6-5*01	2*02	2-7*01	CASSYSGAGVFYEQYF	4.0
8-4*01	32*02	CALDPPDPGGATNKLIF	20-1*01	2*01	2-1*01	CSARLAYSYNEQFF	4.0
9-2*03	6*01	CALSAASGGSYIPTF	20-1*01	2*02	2-1*01	CSASTSGRAVLFNEQFF	4.0
14/DV4*02	20*01	CAMRGTDDYKLSF	29-1*01	2*02	2-7*01	CSVQGTSGSFSYEQYF	4.0

VK10

TRAV	TRAJ	CDR3α	TRBV	TRBD	TRBJ	CDR3β	%
10*01	57*01	CVVSHLTQGGSEKLVF	2*01	2*02	2-7*01	CASSEGGRAYEQYF	33.3
21*02	39*01	CASFGAGNMLTF	11-3*04	2*01	2-7*01	CASSGTSGDEQYF	20.0
38-2/DV8*01	54*01	CAYRSAFWGAQKLVF	12-4*01	2*01	2-5*01	CASSFLAGPGETQYF	13.3
12-1*01	41*01	CVVNMLDSGYALNF	6-4*01	1*01	2-1*01	CASSEGQGYEQFF	6.7
8-4*01	47*02	CAVSVNGNKLVF	10-3*03	2*01	2-7*01	CAISENHGYEQYF	6.7
16*01	48*01	CALIGGNEKLTF	2*01	1*01	1-6*01	CASSPGTGRSPFHF	6.7
3*01	13*02	GLLNVSPGGYQKVTF	5-6*01	2*01	2-1*01	CASSPNSGEQFF	6.7
8-1*01	34*01	CALESYNTDKLIF	20-1*01	1*01	1-3*01	CSARVDIDGNTIYF	6.7

VK19

TRAV	TRAJ	CDR3α	TRBV	TRBD	TRBJ	CDR3β	%
12-3*01	44*01	CAFTGTASKLTF	7-8*01	2*02	2-1*01	CASSPELNEQFF	25.0
12-1*01	12*01	CVVNVPMDSSYKLIF	6-1*01	1*01	2-7*01	CASSEGTGYEQSF	8.3
12-1*01	12*01	CVVNMMDSSYKLIF	2*01	1*01	2-7*01	CASSESQGYEQYF	8.3
12-1*01	30*01	CVVNIRLDDKIIF	2*01	2*02	2-7*01	CASSEGGRAYEQYF	8.3
13-1*02	42*01	CAASMGYGGSQGNLIF	19*01	1*01	2-7*01	CASSMWLGYEQYF	8.3
8-1*01	31*01	CPAHNNARLMF	2*01	2*02	2-7*01	CASSENGGYEQYF	8.3
10*01	40*01	CVVSSLTTSGTYKYIF	7-6*01	1*01	2-7*01	CASSRTGAGEQYF	8.3
13-1*02	28*01	CAASAGSYQLTF	2*01	1*01	2-7*01	CASLGQETVDEQYF	8.3
26-1*01	48*01	CILHSSNFGNEKLTF	5-1*01	1*01	2-3*01	CASSGGQGLSTDTQYF	8.3
27*01	42*01	CAGADYGGSQGNLIF	2*01	2*01	2-2*01	CATRLARTGELFF	8.3

VK42

TRAV	TRAJ	CDR3α	TRBV	TRBD	TRBJ	CDR3β	%
26-1*01	52*01	CIVSPGTSYGKLTF	9*01	2*02	1-1*01	CASSAPVEGNTEAFF	17.6
12-1*01	18*01	CAVILGRGSTLGRLYF	5-1*01	1*01	1-2*01	CASSLEWADNYGYTF	11.8
8-6*02	41*01	CAVRRNSGYALNF	6-1*01	2*01	2-7*01	CASSESGGYEQYF	11.8
12-1*01	20*01	CAVNIPRSNDYKLSF	2*01	1*01	2-7*01	CASSENRGYEQYF	5.9
12-1*01	8*01	CVVADPALVF	2*01	1*01	2-7*01	CASSPWQNRVSSYEQYF	5.9
19*01	4*01	CALRGSGGYNKLIF	6-1*01	1*01	2-7*01	CASSDTGGYEQYF	5.9
2*01	33*01	CAVEDLNSNYQLIW	6-1*01	2*01	2-7*01	CASSEGLGYEQYF	5.9
4*01	5*01	CLVGDMDGTDGRRALTF	6-4*01	2*02	2-7*01	CASSEGVGYEQYF	5.9
34*01	47*02	CGADRYGNKLVF	5-6*01	1*01	1-1*01	CASSLGQGTEAFF	5.9
38-2/DV8*01	22*01	CAYLSPSGSARQLTF	10-3*01	2*01	2-5*01	CAISELGDPGGETQYF	5.9
21*01	31*01	CAVMELNARLMF	27*01	2*01	1-5*01	CASSLSRGQPQHF	5.9
12-2*02	4*01	CAVAGGYNKLIF	27*01	2*01	2-2*01	CASSPSSRNTGELFF	5.9
12-3*01	47*02	CAPYNNGNKLVF	4-1*01	1*01	2-5*01	CASSQGQGQETQYF	5.9

Supplemental Figure S2. TCR pairings of NF9 specific T-cells from vaccinated donors. Variable (V), joining (J), diversity (D) and CDR3 characteristics of NF9 specific TCR pairs. V-J genes colored according to circus plots used elsewhere in the study. The frequency of the TCR pairs are displayed. CDR3β motif CASSX^{3}GYEQYF, and motif-like CDR3s CASSX$^{2-8}$(G)YEQYF, are indicated.

GV38

TRAV	TRAJ	CDR3α	TRBV	TRBD	TRBJ	CDR3β	%
12-2*02	32*02	CAVELGGGATNKLIF	5-4*01	2*01	2-5*01	CASSPRGGQETQYF	6.9
13-1*02	12*01	CAASWSSYKLIF	12-4*01	1*01	2-3*01	CASSRPETGYDTQYF	6.9
12-1*01	28*01	CVVNILYSGAGSYQLTF	6-1*01	1*01	2-7*01	CASSEAKGYEQYF	3.4
12-1*01	28*01	CVVNAVAAGSYQLTF	5-4*01	1*01	2-7*01	CASSIGQGYEQYF	3.4
12-1*01	28*01	CVVILLSGAGSYQLTF	5-4*01	-	1-3*01	CASSLFMDTIYF	3.4
12-1*01	48*01	CVVTRISNFGNEKLTF	10-2*01	1*01	2-5*01	CASTKGWETQYF	3.4
12-3*01	18*01	CLALADRGSTLGRLYF	6-2*01	1*01	2-7*01	CASSTGQGYEQYF	3.4
39*01	53*01	CAVDGNSGGSNYKLTF	7-2*01	2*01	2-7*01	CASSLGLTGYEQYF	3.4
8-3*02	49*01	CAVGYSHLRITGNQFYF	6-4*01	2*02	2-7*01	CASSEAGGYEQYF	3.4
17*01	11*01	CATDSPGYSTLTF	4-3*01	1*01	1-2*01	CASHKFRGANYGYTF	3.4
17*01	54*01	CATDVRTQKLVF	2*01	2*01	2-7*01	CASRPWGTSDYEQYF	3.4
14/DV4*01	40*01	CAMSRSGTYKYIF	9*01	1*01	2-3*01	CASSAPRGGQSTDTQYF	3.4
8-3*02	53*01	CAVDPNSGGSNYKLTF	11-3*04	2*01	2-1*01	CASSDLAGSNEQFF	3.4
14/DV4*01	30*01	CAMRELRDDKIIF	25-1*01	1*01	1-2*01	CASSESYTVYGYTF	3.4
21*01	49*01	CAVEANTGNQFYF	6-2*01	-	2-7*01	CASSHAYEQYF	3.4
19*01	3*01	CARAYSSASKIIF	13*01	1*01	2-1*01	CASSLRDSYNEQFF	3.4
17*01	42*01	CATFYGGSQGNLIF	27*01	1*01	1-2*01	CASSLSDRGAYGYTF	3.4
1-1*01	40*01	CAARTTSGTYKYIF	19*01	2*01	1-2*01	CASSMEGGQFPYGYTF	3.4
12-3*01	5*01	CATNSMDTGRRALTF	6-5*01	1*01	2-3*01	CASSPWTGNTDTQYF	3.4
13-1*02	17*01	CAASMEAAGNKLTF	4-1*01	2*01	2-1*01	CASSQEGLANNEQFF	3.4
8-1*02	16*02	FRAPCSCKDDHKLMF	4-1*01	1*01	2-2*01	CASSQGVVLQGELFF	3.4
13-2*01	52*01	CAESPNAGGTRYGKLTF	4-1*01	2*02	2-1*01	CASSQWSGSNYEQFF	3.4
8-1*01	39*01	CAVTLLNAGNMLTF	5-5*02	1*01	2-7*01	CASSRTQYYEQYF	3.4
12-2*02	28*01	CAVMSYSGAGSYQLTF	9*01	2*01	2-7*01	CASSVASGAYEQYF	3.4
27*01	38*01	CAGPHAGNNRKLIW	6-6*01	1*01	1-5*01	CASSYLGGPSNQPQHF	3.4
14/DV4*02	24*03	CAMREREATDSWGKFQF	30*01	1*01	1-4*01	CAWSPGFPPNEKLFF	3.4
13-1*02	8*01	CAASRVDTGFQKLVF	20-1*01	2*01	2-3*01	CSARSPIVTDTQYF	3.4

GV41

TRAV	TRAJ	CDR3α	TRBV	TRBD	TRBJ	CDR3β	%
12-1*01	6*01	CVVNGWEGGSYIPTF	20-1*01	2*01	2-3*01	CSARDRQADDTDTQYF	5.6
21*01	18*01	CAVRSDRGSTLGRLYF	11-2*03	1*01	2-7*01	CASSLGQGYEQYF	5.6
14/DV4*02	11*01	CAFPFSGYSTLTF	27*01	1*01	2-7*01	CASSLWQGYEQYF	5.6
12-2*01	42*01	CAVLNYGGSQGNLIF	10-3*03	2*01	2-7*01	CAISEGRAYEQYF	5.6
4*01	42*01	CLVIYGGSQGNLIF	4-1*01	2*02	2-7*01	CASSQGESYEQYF	5.6
4*01	22*01	CLVWGSARQLTF	2*01	1*01	2-7*01	CASSEGTGGYEQYF	5.6
4*01	10*01	CLVGDMNRFTGGGNKLTF	5-6*01	1*01	2-7*01	CASSLGGRGYEQYF	5.6
3*01	47*02	CAVRDDSYGNKLVF	5-6*01	2*02	2-3*01	CASSLEVGKWTDTQYF	5.6
8-6*01	4*01	CATSGGYNKLIF	28*01	1*01	1-1*01	CASSPRTGWTEAFF	5.6
17*01	15*01	CATGLNQAGTALIF	29-1*01	2*02	1-3*01	CSVEVTAPGEGGNTIYF	5.6
38-2/DV8*01	41*01	QAYRSTGYAETL	29-1*01	1*01	2-7*01	CSVVGVRPDEQYF	5.6
19*01	29*01	CALSESNSGNTPLVF	24-1*01	1*01	2-7*01	CATSAVGHSGNEQYF	5.6
3*01	23*01	CAVRFPGNQGGKLIF	28*01	2*01	2-1*01	CASRPGLAGDNEQFF	5.6
21*01	44*01	PQAGTASKLTF	20-1*01	1*01	2-7*01	CSASRLGTGSYEQYF	5.6
8-3*02	41*01	CAVGLSGYALNF	19*01	1*01	2-7*01	CASSIERRTSDYEQYF	5.6
6*02	43*01	CALDNNNNDMRF	28*01	2*01	2-7*01	CASRLRDWDDEQYF	5.6
4*01	5*01	CLVGDTRPQDTGRRALTF	4-2*01	2*01	2-3*01	CASSQGSGGDTQYF	5.6
29/DV5*04	20*01	CAARLSNDYKLSF	4-1*01	1*01	2-1*01	CASWDANRNEQFF	5.6

KK-008

TRAV	TRAJ	CDR3α	TRBV	TRBD	TRBJ	CDR3β	%
12-1*01	41*01	CVVNMLRNSGYALNF	6-1*01	2*01	2-7*01	CASSESGGYEQYF	22.5
26-1*02	23*01	CIGAYNQGGKLIF	2*01	2*01	2-7*01	CASSENRGYEQYF	9.8
12-1*01	12*01	CVVNIIMDSSYKLIF	6-4*01	2*02	2-7*01	CASSEGEGYEQYF	8.8
12-1*01	33*01	CVVNEYRSSNYQLIW	5-4*01	1*01	2-7*01	CASSLGQGYEQYF	8.8
12-1*01	12*01	CVVNLMDSSYKLIF	2*01	1*01	2-7*01	CASSVSQGYEQYF	6.9
1-1*01	30*01	CAVRGKNRDDKIIF	20-1*01	1*01	2-3*01	CSARPRDWGLGTDTQYF	5.9
12-1*01	12*01	CVVNMLLDSSYKLIF	4-1*01	1*01	2-1*01	CASSDTQGYEQFF	4.9
12-1*01	54*01	CVVNTPIQGAQKLVF	2*01	1*01	2-7*01	CASSENRKDPNSC	4.9
12-1*01	33*01	CVVIIRGDSNHQLTW	7-3*01	1*01	2-7*01	CASSYGQGYEQYF	3.9
12-1*01	28*01	CVVNRLGAGSYQLTF	7-2*02	1*01	2-7*01	CASSSGQGYEQYF	3.9
38-1*01	40*01	CAFFLEGTYKYIF	20-1*01	2*02	2-7*01	CSVGASGSYEQYF	3.9
12-1*01	12*01	CVVNRMDSSYKLIF	10-3*03	1*01	2-7*01	CAISEQQGYEQYF	3.9
24*01	53*01	CAFPVGGSNYKLTF	20-1*01	2*01	2-7*01	CSARDISGGGQYEQYF	3.9
26-1*02	37*02	CIVMGSSNTGKLIF	2*01	1*01	2-7*01	CASSEFRTYEQYF	2.9
12-1*01	28*01	CVVNIHSGAGSYQLTF	5-4*01	1*01	2-7*01	CASSIGQGYEQYF	2.0
12-1*01	28*01	CVVNKHSGAGSYQLTF	5-4*01	1*01	2-7*01	CASSQGQGYEQYF	2.0
20*02	57*01	CVRYLTPQGGSEKLVF	2*01	1*01	2-7*01	CASSEFQGYEQYF	1.0

GV42

TRAV	TRAJ	CDR3α	TRBV	TRBD	TRBJ	CDR3β	%
5*01	24*02	CAEVVNDSWGKLQF	2*01	1*01	2-7*01	CASSESQGYEQYF	4.9
19*01	7*01	CALSEATGGGNNRLAF	5-5*02	1*01	1-5*01	CASSLGWGNQPQHF	3.3
12-1*01	9*01	CVVNMWGTGDFKIIF	27*01	1*01	2-1*01	CASGALGNEQFF	3.3
12-1*01	12*01	CVVNLLMDSSYKLIF	2*01	1*01	2-4*01	CASSEGQGYIQYF	1.6
12-1*01	43*01	CVVTPVPNDMRF	6-1*01	1*01	2-7*01	CASSEGQSYEQYF	1.6
12-1*01	12*01	CVVNALEDSSYKLIF	2*01	1*01	2-7*01	CASSAGQGYEQYF	1.6
12-1*01	12*01	CVVNIFLDSSYKLIF	2*01	2*01	2-7*01	CASSATAGYEQYF	1.6
12-1*01	40*01	CVVSPPGPSGTYKYIF	2*01	1*01	2-7*01	CASSDFQGYEQYF	1.6
12-1*01	42*01	CVVTPNYGGSQGNLIL	2*01	1*01	2-7*01	CASSEYMSYEQYF	1.6
12-1*01	34*01	CVVQGDTDKLIF	6-1*01	1*01	2-7*01	CASSDWVGYEQYF	1.6
12-2*01	53*01	CAVNGLAGGSNYKLTF	6-1*01	1*01	2-7*01	CASSEGSGYEQYF	1.6
12-1*01	12*01	CVVNRLMDSSYKLIF	6-1*01	2*01	2-7*01	CASSVAKGYEQYF	1.6
12-2*01	28*01	CASSRPSSGAGSYQLTF	6-1*01	1*01	2-7*01	CASSEWGQGGYEQYF	1.6
12-1*01	20*01	CVVSGDYKLSF	2*01	2*01	2-7*01	CASSEFAGKGSSYEQYF	1.6
12-1*01	5*01	CVVNIGTGRRALTF	4-1*01	1*01	2-7*01	CASSLGQDYEQYF	1.6
12-1*01	12*01	CVVTRWMDSSYKLIF	5-6*01	2*01	2-1*01	CASSHWTLQPDNEQFF	1.6
12-1*01	8*01	CVVNGRWNANTGFQKLVF	9*01	2*01	2-7*01	CASSVAWTSVYEQYF	1.6
38-2/DV8*01	39*01	CAYHNNAGNMLTF	11-2*01	1*01	2-7*01	CASSRWQNYEQYF	1.6
20*02	10*01	CAVQTGGGNKLTF	6-4*01	2*01	1-2*01	CASSDGLGYTF	1.6
34*01	21*01	CGAASPGGKFYF	2*01	1*01	2-7*01	CASSEYTPLGHEQYF	1.6
8-3*01	42*01	CAVGIYGGSQGNLIF	12-4*01	2*01	2-1*01	CASSFGSGAYNEQFF	1.6
4*01	43*01	CLVGDSDDMRF	7-9*03	1*01	1-1*01	CASSFRGTEAFF	1.6
16*01	49*01	CALTSGNQFYF	4-2*01	2*02	2-7*01	CASSHMSSGNYEQYF	1.6
8-4*01	22*01	CAVSDVYGSARQLTF	19*01	2*01	2-3*01	CASSIGGRTTDTQYF	1.6
38-1*03	36*01	CAFIGLTGANNLFF	19*01	2*02	2-5*01	CASSILSGRTGGETQYF	1.6
8-3*01	47*01	CAVVPMEYGNKLVF	7-9*03	1*01	2-3*01	CASSLALRQGATDTQYF	1.6
41*01	32*02	CAVRPGYGGATNKLIF	5-4*01	2*01	2-3*01	CASSLALTDTQYF	1.6
24*01	6*01	CASRRLSGGSYIPTF	2*01	1*01	2-3*01	CASSLDWGTAHTDTQYF	1.6
14/DV4*01	39*01	CSMIEDNTDTMLTF	7-9*03	2*02	1-1*01	CASSLGGGKVNTEAFF	1.6
9-2*04	49*01	CAPPNTGNQFYF	13*01	1*01	2-6*01	CASSLGQGQGYSGANVLTF	1.6
16*01	10*01	CARRGGNKLTF	7-3*01	1*01	2-3*01	CASSLSGGGTDTQYF	1.6
8-6*02	9*01	CAVDTGGFKTIF	7-9*03	1*01	1-2*01	CASSLTRTLPKGQLYGYTF	1.6
20*02	39*01	CAVPNNNAGNMLTF	4-1*01	2*02	2-7*01	CASSQDFSGSYEQYF	1.6
8-1*01	5*01	CAVFPWAGRRALTF	14*01	2*02	1-1*01	CASSQGGGDTEAFF	1.6
12-3*01	34*01	CAMSHYNTDKLIF	5-1*01	-	2-5*01	CASSSEETQYF	1.6
21*02	18*01	CAVQGRGSTLGRLYF	9*01	1*01	2-5*01	CASSSEGGVGETQYF	1.6
8-4*03	49*01	CAVTFSNQFYF	7-9*03	2*01	1-1*01	CASSSRGATGTEAFF	1.6
22*01	12*01	CAVWMDSSYKLIF	9*01	1*01	1-1*01	CASSVAGVTEAFF	1.6
12-2*02	35*01	CAVNMPPIGFGNVLHC	9*01	2*01	2-1*01	CASSVGPGVGNEQFF	1.6
14/DV4*02	42*01	CALVRYYGGSQGNLIF	6-4*01	2*02	2-1*01	CASSVSGSEGHEQFF	1.6
29/DV5*04	43*01	CAATWGDMRF	5-1*01	2*01	2-3*01	CASSYWGGSDTQYF	1.6
13-1*02	21*01	CAAGSTLYNFNKFYF	4-1*01	1*01	1-1*01	CASTGRAGGTEAFF	1.6
13-1*02	43*01	CAASMRDNDMRF	15*02	1*01	2-7*01	CATSKESGAPYEQYF	1.6
3*01	49*01	CAVRDTGNQFYF	30*01	1*01	1-2*01	CAWNHGGLYGYTF	1.6
5*01	40*01	CAVTSGTYKYIF	30*01	1*01	2-1*01	CAWSPLKGREQFF	1.6
12-3*01	53*01	CAIGGSNYKLTF	20-1*01	2*02	2-1*01	CSAHGGPTSGSFIEQFF	1.6
8-4*01	10*01	CAVTLHGGGNKLTF	20-1*01	2*02	2-3*01	CSAPTEERTDTQYF	1.6
22*01	20*01	CAVERKQTSSAF	20-1*01	1*01	2-1*01	CSARAGETFSSYNEQFF	1.6
19*01	13*02	CALSERNSGGYQKVTF	20-1*01	1*01	2-3*01	CSARARQDHTDTQYF	1.6
38-2/DV8*01	30*01	CAYRSARDDKIIF	20-1*01	1*01	2-7*01	CSAREQDYEQYF	1.6
13-2*01	39*01	CAENSYNNAGNMLTF	20-1*01	2*01	2-1*01	CSARPLAASSYNEQFF	1.6
12-2*01	45*01	CAVNIGSGGGADGLTF	29-1*01	1*01	2-3*01	CSAWDRFTDTQYF	1.6
38-1*03	39*01	CAFMKPNAGNMLTF	20-1*01	1*01	1-3*01	CSGMDGSGNTIYF	1.6
19*01	41*01	CALSTSNSGYALNF	29-1*01	2*02	2-1*01	CSVEGLAGEWEQFF	1.6
17*01	3*01	CATHLRGSSASKIIF	29-1*01	2*01	2-5*01	CSVVLAENAEETQYF	1.6
12-2*01	53*01	CAVNDSGGSNYKLTF	20-1*01	1*01	2-7*01	CSSVSISSYEQYF	1.6
4*01	9*01	CLTPYTGGFKTIF	28*01	1*01	1-6*01	CASIPYQDRGLFEMNSPLHF	1.6

Supplemental Figure S3. TCR pairings of NF9 specific T-cells from convalescent donors. Variable (V), joining (J), diversity (D) and CDR3 characteristics of NF9 specific TCR pairs. V-J genes colored according to circus plots used elsewhere in the study. The frequency of the TCR pairs are displayed. CDR3β motif CASSX^{3}GYEQYF, and motif-like CDR3s CASSX$^{2-8}$(G)YEQYF, are indicated.

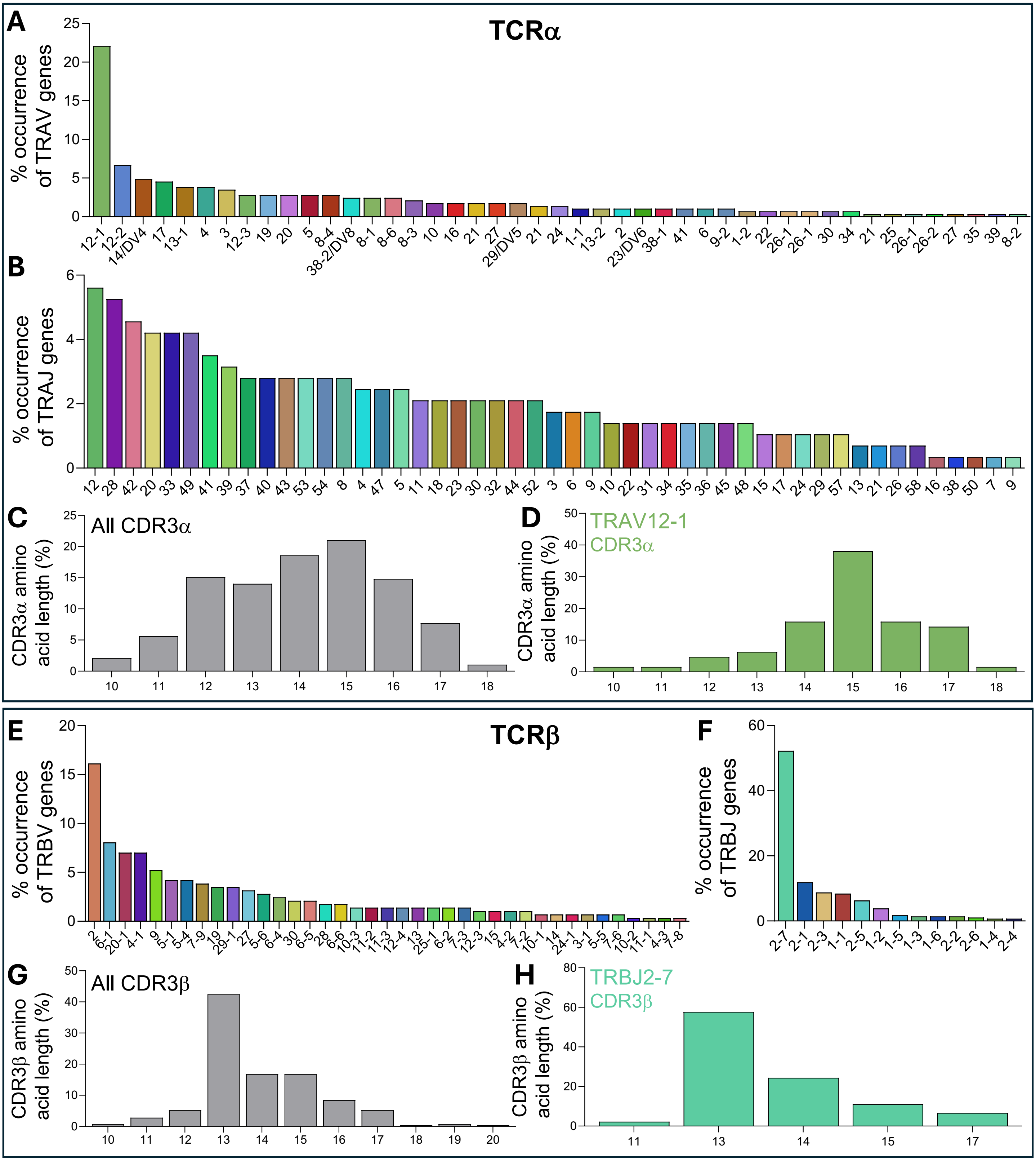

Supplemental Figure S4. TCRα and TCRβ characteristics of NF9-specific T-cell responses in vaccinated and convalescent donors. (**A**) TCRα V-gene usage. (**B**) TCRα J-gene usage. (**C**) CDR3α length. (**D**) CDR3α length of TRAV12-1 containing CDR3α. (**E**) TCRβ V-gene usage. (**F**) TCRβ J-gene usage. (**G**) CDR3β length. (**H**) CDR3β length of TRBJ2-7 containing CDR3α.

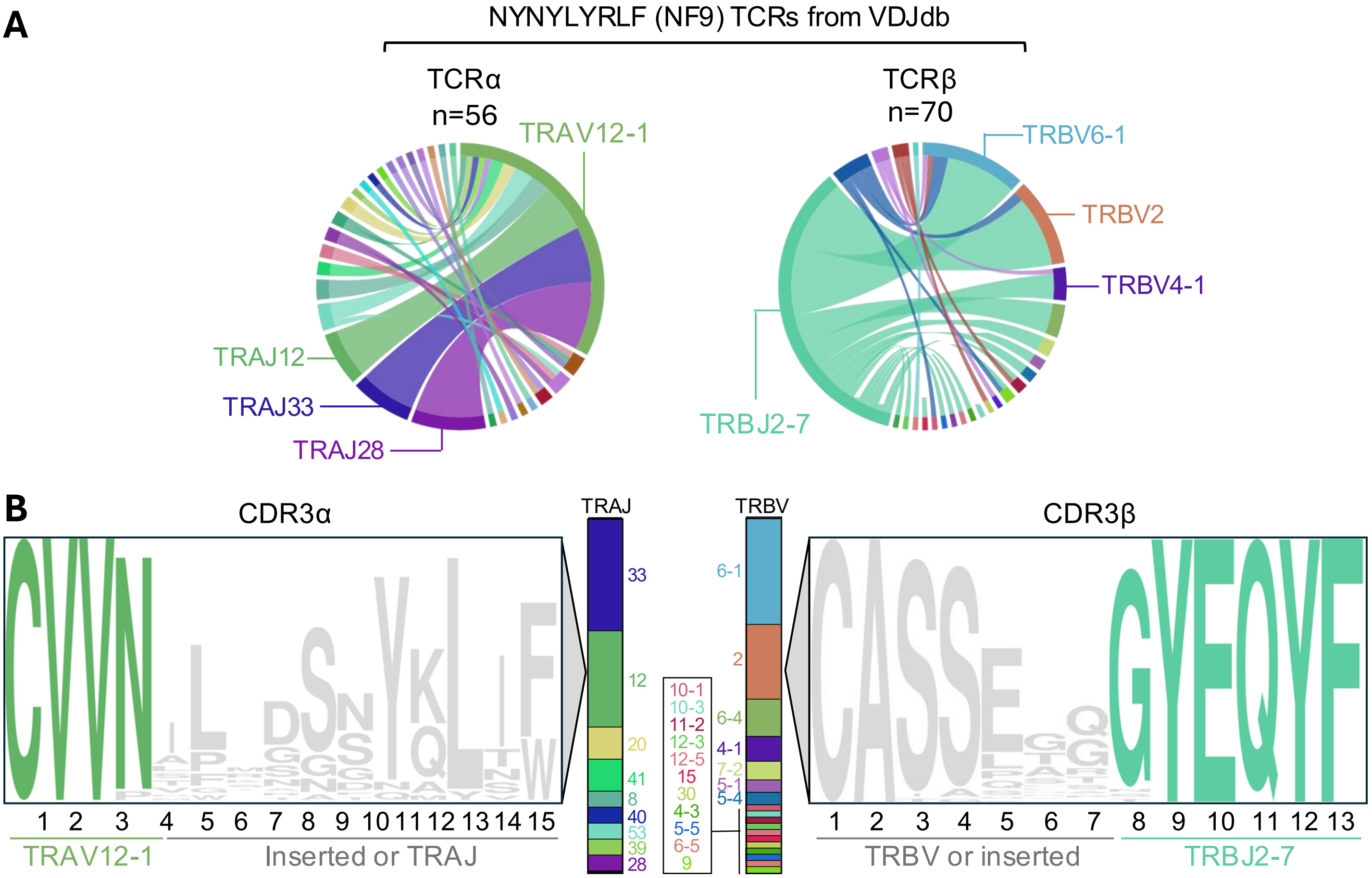

Supplemental Figure S5. Clonotypic analysis of NYNYLYRLF-containing CDR3 sequences from VDJdb. Data taken from VDJdb for HLA-A*24:02 NYNYLYRLF specific TCRs including CDR3s with TRAV-TRAJ (56 chains) and TRBV-TRBJ (70 chains) gene usage. (**A**) Circos plots show the proportion of TRAJ or TRBJ genes on the left and TRAV or TRBV genes on the right, with the size of the arcs corresponding to relative frequency of the genes. Ribbons between the arcs represents V-J pairings. (**B**) Logo plots of CDR3α (left) and CDR3β (right) from VDJdb TCRs in (**A**). CDR3α: based on TRAV12-1 (most prevalent TRAV) of 15 amino acids in length. CDR3β motif: based on TRBJ2-7 and 13 amino acids in length. The multiple TRAJ and TRBV chains that contribute to the CDR3 motifs are shown in central bars, where they are ordered from highest to lowest frequency.

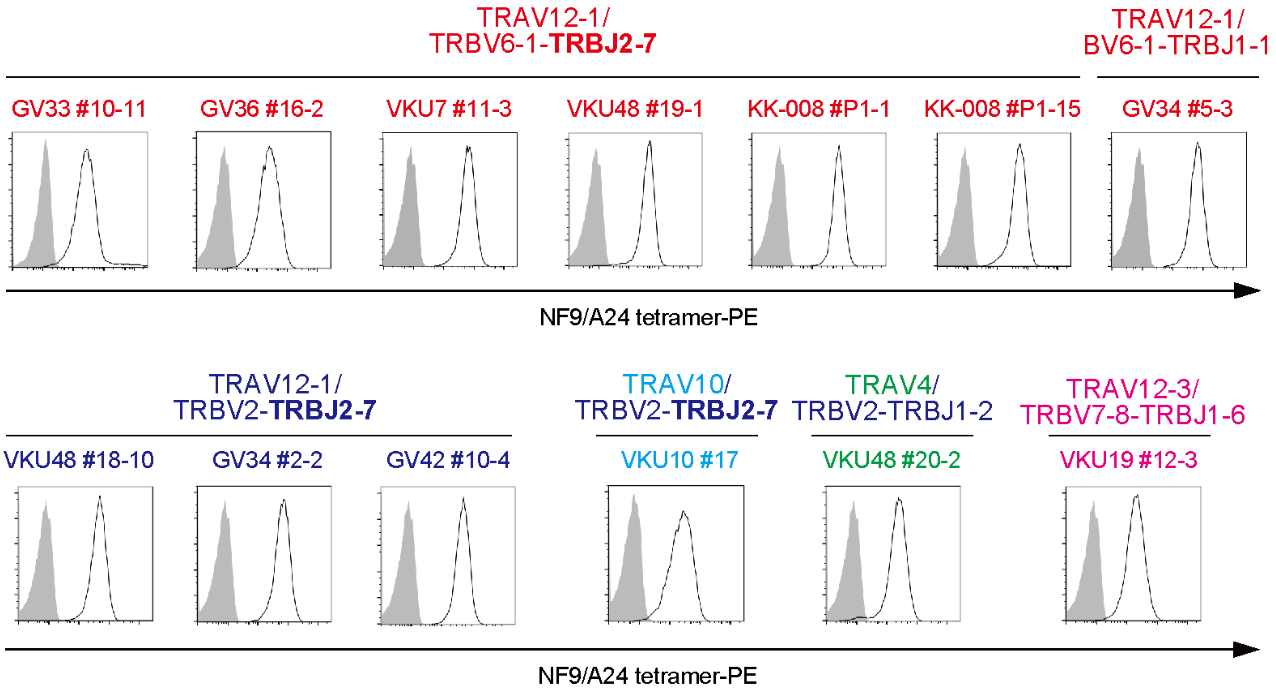

Supplemental Figure S6. A24/NF9 tetramer staining of TCR-transduced Jurkat cells. A Jurkat cells alone (shaded histogram) or those expressing A24/NF9-specific TCRs (GV33 #10-11, GV34 #5-3, GV36 #16-2, VKU7 #11-3, VKU48 #19-1, KK-008 #P1-1, and KK-008 #P1-15, VKU48 #18-10, GV34 #2-2, GV42 #10-4, VKU #20-2, VKU #17 and VKU #12-3) (open histogram) were stained with anti-CD3 mAb and A24/NF9 tetramer and then analyzed by flow cytometry.

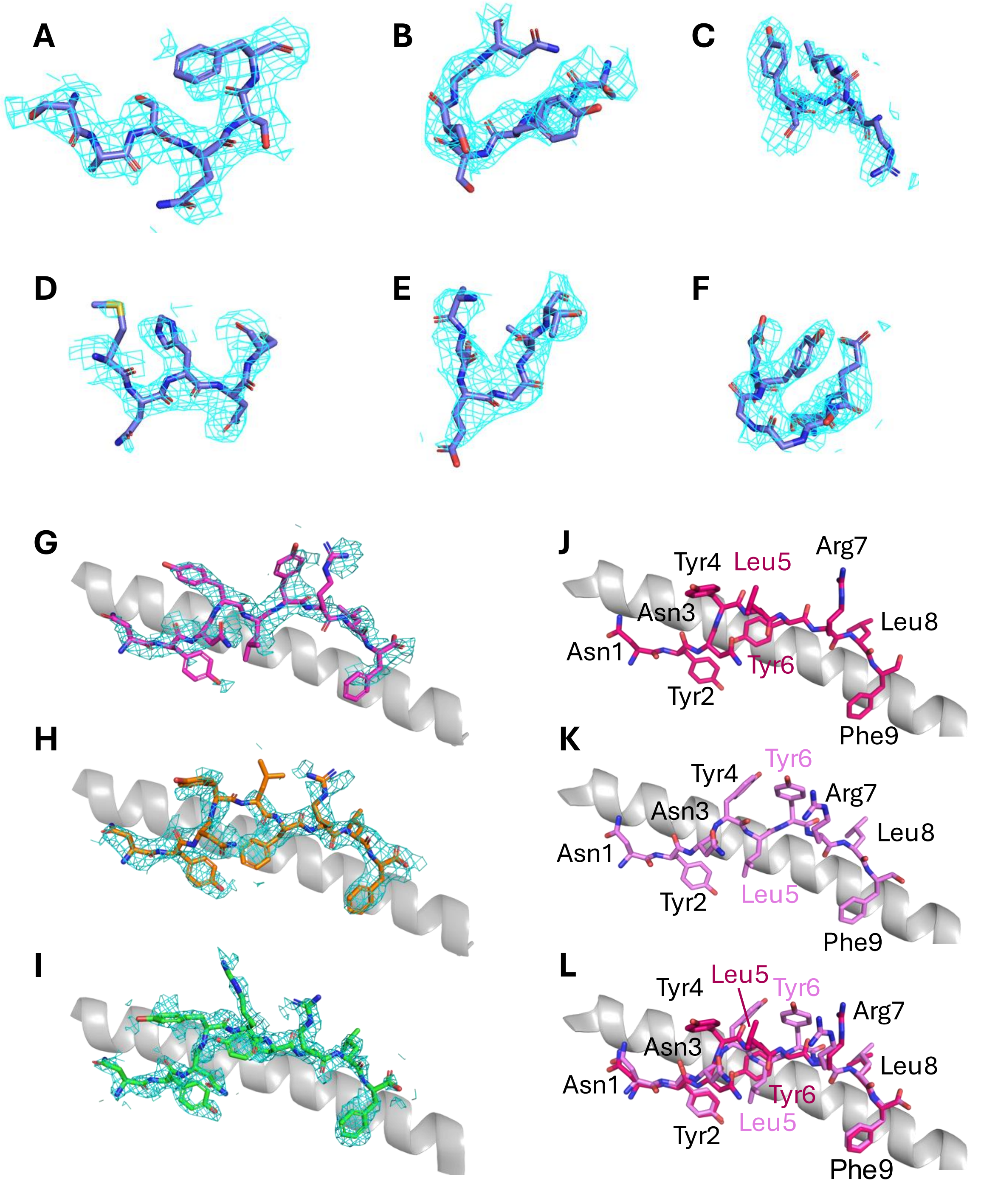

Supplemental Figure S7. OMIT maps confirming robustness of X-ray crystallography models and Conformational change of the NF9 peptide and TCR cross-reactivity (A-F) 3D structural maps of P1-15 Complementary Determining Regions (CDR) Loops (**A** – CDR1α, **B** – CDR2α, **C** – CDR3α, **D** – CDR1β, **E** – CDR2β, **F** – CDR3β) accompanied by their respective electron densities (cyan mesh). Unbiased omit maps were calculated after removal of the loops from the model to minimise model bias. (**G–I**) 3D structural maps of the NF9 peptide in complex with the P1-15 TCR (magenta sticks), NF9-6F peptide (orange sticks), and NF9-5R peptide (green sticks), accompanied by their respective electron densities (cyan mesh). Unbiased omit maps were calculated after removal of the peptide from the model to minimise model bias. The resulting electron density supports the assigned peptide backbone conformations and clearly resolves the distinct P5 and P6 orientations described in the main text. (**J**) Presentation of copy 1 of the NF9 peptide when not in complex with TCR, published by Zhang et al. (PDB 7F4W). (**K**) As in (A) but copy 2 published by Zhang et al. (PDB 7F4W). (**L**) Comparison of copy 1 and 2 from (A) and (B).

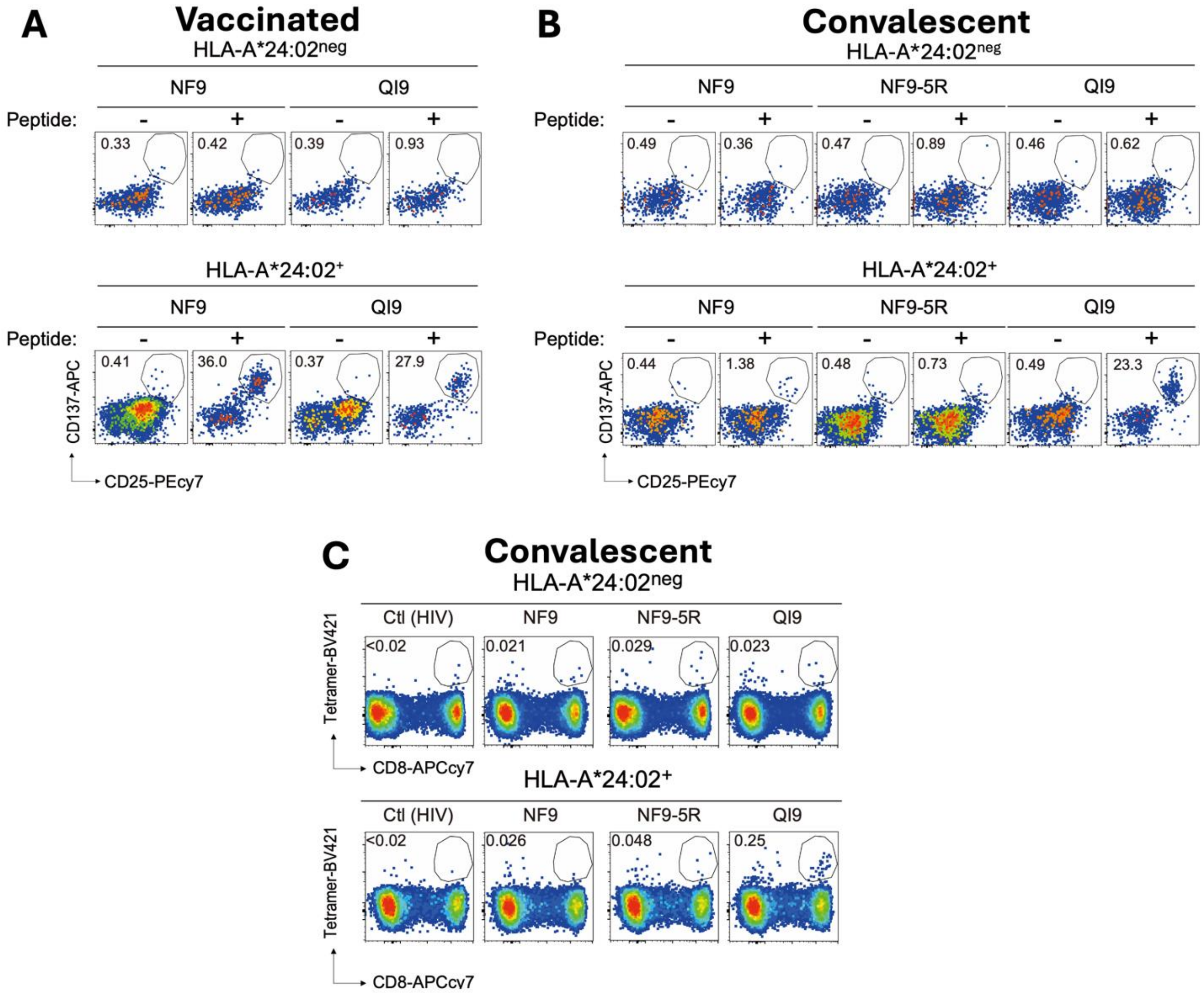

Supplemental Figure S8. NF9 5R specific T-cells are not detectable in convalescent donors infected with the SARS-CoV-2 Delta variant: example flow cytometry plots. (**A**) PBMCs from vaccinated donors stimulated with NF9 and QI9 peptides. Example flow cytometry plots shown for an HLA-A*24:02^{neg} or HLA-A*24:02^{+} donor. (**B**) PBMCs from convalescent donors infected with the delta strain. Example flow cytometry plots shown for an HLA-A*24:02^{neg} or HLA-A*24:02^{+} donor. (**C**) Tetramer staining of PBMCs from HLA-A*24:02 negative (n = 6) or positive (n = 8) convalescent donors infected with the delta strain. Example flow cytometry plots shown for an HLA-A*24:02^{neg} or HLA-A*24:02^{+} donor.
